# Outcomes of controlled human malaria infection after BCG vaccination

**DOI:** 10.1038/s41467-019-08659-3

**Published:** 2019-02-20

**Authors:** Jona Walk, L. Charlotte J. de Bree, Wouter Graumans, Rianne Stoter, Geert-Jan van Gemert, Marga van de Vegte-Bolmer, Karina Teelen, Cornelus C. Hermsen, Rob J. W. Arts, Marije C. Behet, Farid Keramati, Simone J. C. F. M. Moorlag, Annie S. P. Yang, Reinout van Crevel, Peter Aaby, Quirijn de Mast, André J. A. M. van der Ven, Christine Stabell Benn, Mihai G. Netea, Robert W. Sauerwein

**Affiliations:** 1 Department of Medical Microbiology, Radboud University Medical Center, PO Box 9101, 6500 HB Nijmegen, The Netherlands; 2 Radboud Center for Infectious Diseases, Radboud University Medical Center PO Box 9101, 6500 HB Nijmegen, The Netherlands; 3 Department of Internal Medicine, Radboud University Medical Center PO Box 9101, 6500 HB Nijmegen, The Netherlands; 4 Research Center for Vitamins and Vaccines, Bandim Health Project, Statens Serum Institut, 2300 Copenhagen, Denmark; 5 Odense Patient Data Explorative Network, University of Southern Denmark/Odense University Hospital, 5000 Odense, Denmark; 6 Department of Molecular Biology, Faculty of Science, Radboud university, 6525 GA Nijmegen, The Netherlands; 7 Department for Genomics & Immunoregulation, Life and Medical Sciences Institute (LIMES), University of Bonn, 53115 Bonn, Germany

## Abstract

Recent evidence suggests that certain vaccines, including Bacillus-Calmette Guérin (BCG), can induce changes in the innate immune system with non-specific memory characteristics, termed ‘trained immunity’. Here we present the results of a randomised, controlled phase 1 clinical trial in 20 healthy male and female volunteers to evaluate the induction of immunity and protective efficacy of the anti-tuberculosis BCG vaccine against a controlled human malaria infection. After malaria challenge infection, BCG vaccinated volunteers present with earlier and more severe clinical adverse events, and have significantly earlier expression of NK cell activation markers and a trend towards earlier phenotypic monocyte activation. Furthermore, parasitemia in BCG vaccinated volunteers is inversely correlated with increased phenotypic NK cell and monocyte activation. The combined data demonstrate that BCG vaccination alters the clinical and immunological response to malaria, and form an impetus to further explore its potential in strategies for clinical malaria vaccine development.

## Abstract (web summary)

Immune activation induces long-term alterations of setpoints, impacting responses to subsequent unrelated stimuli. Here the authors show that volunteers vaccinated with BCG respond to controlled human malaria infection with increased clinical symptoms and an inverse correlation between immune activation markers and parasitemia.

## Introduction

With nearly 200 million clinical cases and nearly half a million deaths in 2015^1^, malaria remains a major global health problem and there is pressing need for a highly efficacious vaccine. RTS,S (Mosquirix®, GlaxoSmithKline), the only registered malaria vaccine, confers only modest, short-term protection^2^. It is clear that novel and improved malaria vaccine strategies are required for eradication.

To date malaria vaccine research has focused primarily on the induction of strong antibody or T-cell responses. However, recent evidence suggests that certain vaccines, including the Bacillus-Calmette Guérin (BCG) developed against tuberculosis, can induce long-term changes in the innate immune system with non-specific memory characteristics. This BCG-induced ‘trained immunity’^3^ increases pro-inflammatory cytokine responses to other pathogens^4,5^ and is mediated by epigenetic changes in innate immune cells^6,7^. The clinical relevance of trained innate immunity has been demonstrated in mice, where it reduced mortality of *Staphylococcus aureus* sepsis^6^ and *Candida albicans* infection^8^.

There is also evidence that BCG administration reduces parasitemia in rodent malaria models^9–12^, and in endemic areas BCG vaccination has been associated with reduced malaria-specific mortality^13^. However, any direct evidence for protective efficacy of BCG-induced trained immunity against malaria, or any clinically relevant pathogen, in humans is lacking.

Here, we show that a subset of BCG vaccinated healthy volunteers have accelerated NK cell and monocyte activation that correlates with reduced parasitemia after controlled human malaria infection (CHMI). These findings are consistent with the possibility that BCG vaccination may induce trained immunity with functional activity against another human pathogen in vivo.

## Results

### BCG vaccination alters the clinical course of *P. falciparum* infection

In a single-blind, randomised controlled clinical trial, ten healthy BCG- and malaria-naive volunteers received an intradermal BCG vaccination, while ten control volunteers received no intervention. A single volunteer was excluded post vaccination due to a concomitant Epstein–Barr virus infection. Five weeks after vaccination, 9 BCG vaccinated and 10 control volunteers underwent a Controlled Human Malaria Infection (CHMI) by exposure to bites of five *P. falciparum* (*Pf*) infected female *Anopheles* mosquitoes (Supplementary Fig. 1). Randomisation was stratified by gender in order to ensure an equal distribution of male and female volunteers. Other baseline characteristics were similar between groups (Supplementary Table 1). Study primary endpoints were (1) Frequency and magnitude of adverse events and (2) Time to blood stage parasitemia detectable by quantitative PCR (qPCR). Study secondary endpoints were (1) Changes in cellular (innate and adaptive) immune responses and (2) Changes in plasma cytokine levels.

**Fig. 1:**
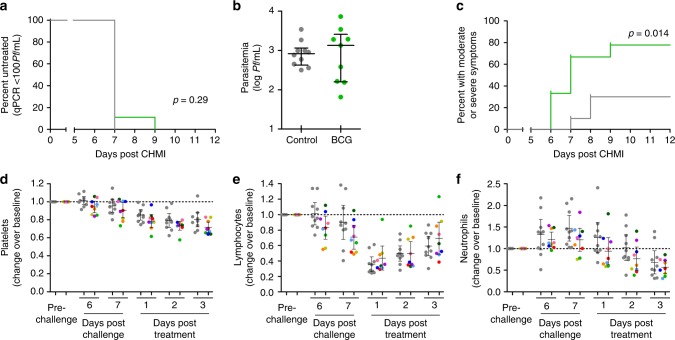
Parasitemia, clinical symptoms and laboratory abnormalities after CHMI. Parasitemia was measured by daily qPCR from day 6 after CHMI until the third day after antimalarial treatment. **a** The Kaplan–Meier survival curve shows percent of volunteers remaining untreated. 8/9 BCG vaccinated (green) and 10/10 control volunteers (grey) surpassed the treatment threshold of 100 parasites per millilitre, and were treated on day 7 after challenge. 1/9 BCG vaccinated volunteers remained below 100 Pf/mL until day 9. **b** All volunteers did have parasitemia detectable by qPCR on day 7 after CHMI. The graph shows log parasites per millilitre on day 7 post CHMI for BCG vaccinated (green) and control (grey) volunteers. **c** Adverse events were collected daily. The Kaplan–Meier curve shows the percentage of volunteers experiencing one or more moderate or severe, solicited, symptoms during follow-up, BCG vaccinated volunteers (green) compared to controls (grey). **d**–**f** Absolute platelet, lymphocyte and neutrophil differentiation counts were determined by daily hemocytometry starting on day 6 post-challenge. Graphs show relative change in cell counts compared to pre-challenge values in both BCG vaccinated (*n* = 9, each coloured dot shows and individual volunteer, colours consistently represent the same volunteers across each graph) and non-BCG vaccinated controls (*n* = 10, grey dots)

All volunteers developed parasitemia as detected by qPCR after challenge infection. Blood samples from 8 out of 9 BCG vaccinated and all controls exceeded the predetermined threshold of 100 parasites per millilitre blood on day 7, which was followed by a curative treatment with atovaqone/proguanil. One BCG vaccinated volunteer became positive on day 9. Interestingly, the variation in day 7 parasitemia was much higher in the BCG vaccinated group (geometric mean: 752 Pf/mL, 95% CI: 217–2602 Pf/mL) than in the controls (geometric mean: 813 Pf/mL, 95% CI 481–1373 Pf/mL) (Levene’s test for equality of variances: *p* = 0.005, Fig. 1a, b).

BCG vaccinated volunteers developed clinical symptoms of malaria infection at an earlier time point and reported a higher frequency of moderate or severe clinical symptoms than control volunteers (Gehan-Breslow-Wilcoxon Test, *p* = 0.01, Fig. 1c; Supplementary table 2). The moderate and severe adverse event frequency in the BCG vaccinated group was also significantly higher than in historical controls (Supplementary Fig. 2). In line with this finding, BCG vaccinated volunteers presented with a more significant decrease in platelet count (mean relative change BCG group: 0.689, 95% CI: 0.637–0.741; control group: 0.778, 95% CI: 0.703–0.853; student’s *t*-test: *p* = 0.05; Supplementary Fig. 3). Moreover, in a subset of BCG vaccinated volunteers, circulating platelets, lymphocytes and neutrophils dropped earlier (Fig. 1d–f). There was no significant difference in temperature during follow-up (Supplementary Fig. 4).

### BCG vaccinated volunteers show memory-like immune responses

**Fig. 2:**
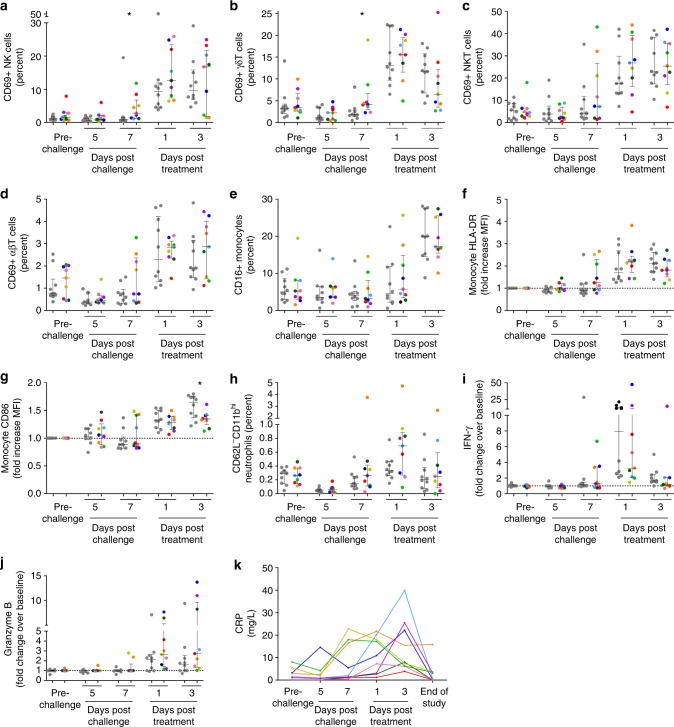
In vivo activation of lymphocytes, monocytes and neutrophils after CHMI. In vivo leucocyte activation was determined by direct staining of fresh whole blood with fluorescent antibodies every 2 days post-challenge. Lymphocytes were defined based on forward scatter and sideward scatter characteristics, and duplet events were excluded. **a** NK cell activation was defined as the percentage of CD3^-^CD56^dim^CD16^+^ live cells expressing CD69. **b** γδT cell activation was defined as the percentage of CD3^+^γδTCR^+^ live cells expressing CD69. **c** NKT cell activation was defined as the percentage of CD3^+^γδTCR^-^CD56^+^ live cells expressing CD69. **d** αβT cell activation was defined the as percentage of CD3^+^γδTCR^-^CD56^−^ live cells expressing CD69. **e** Monocytes were defined based on forward and side scatter characteristics, and the as HLA-DR^+^CD14^+^. Within the monocyte population, cells were then divided into CD16^-^ and CD16^+^ monocytes. **f**–**g** Within the CD16^-^ monocyte population, the relative change in mean fluorescent intensity of HLA-DR and CD86 compared to pre-malaria challenge values was determined. **h** Neutrophils were defined based on forward and side scatter characteristics, and then defined as HLA-DR^-^CD14^-^CD16^+^CD11b^+^. Activated neutrophils were defined as CD62L^dim^CD11b^high^. **i**–**j** IFN-γ and granzyme B were measured by Luminex assay in citrate plasma taken ever 2 days. Circulating cytokine levels are corrected for baseline levels (pre-BCG vaccination time point) at each time point. In all graphs the grey dots represent non-BCG vaccinated control group volunteers (*n* = 10), and each coloured dot shows an individual BCG vaccinated volunteer (*n* = 9). Statistical analysis are between BCG vaccinated and control volunteers at a single time point, and *p*-values are the results of Mann–Whitney *U*-test. *p < 0.05. **k** Circulating CRP levels were measured in citrate plasma are shown for each BCG vaccinated volunteer (colours consistently represent the same volunteers across each graph)

To study the kinetics of the immune response to CHMI, whole blood flow cytometry was performed to determine lymphocyte, monocyte and neutrophil activation. Post-BCG vaccination but prior to CHMI and during parasite liver stage (day 5 post-challenge), there was no activation of peripheral blood leucocytes in either group as measured by the expression of the early activation marker CD69 in lymphocytes, or the expression of CD16, the antigen presenting molecule HLA-DR, and the co-stimulatory molecule CD86, in monocytes (Fig. 2a–g). On day 7, coinciding with the first appearance of blood stage parasites, there was a marked increase in the proportion of CD56^dim^ NK cells expressing CD69 in half of the BCG vaccinated volunteers, absent in the control group (Mann–Whitney *U*-test of vaccinated vs. controls: *p* = 0.03, Fig. 2a). Instead, control volunteers primarily showed immune activation only after treatment. CD69 expression on gamma-delta (γδ) T cells, NKT cells and alpha-beta (αβ) T cells followed a similar pattern, with the same subgroup of BCG vaccinees showing activation on day 7 post-challenge (Fig. 2b–d). There was no significant increase in CD69 expression on CD56^bright^ NK cells after challenge (Supplementary Fig. 5).

Next, the expression of CD16, HLA-DR and CD86, were determined on CD14^+^ monocytes (Fig. 2e–g), markers previously shown to increase with monocyte activation during CHMI^14^. The percentage of activated neutrophils, those lacking CD62L with high CD11b expression, was also analysed (Fig. 2h). On day 7 after challenge, the same subgroup of BCG vaccinated volunteers showed increased expression of HLA-DR and CD86 on CD14^+^CD16^−^ monocytes, which was absent in controls.

The three BCG vaccinees with the strongest lymphocyte and monocyte activation also responded with early increases in plasma interferon-gamma (IFN-γ) or granzyme B, and inflammatory C-reactive protein (CRP) concentrations (Fig. 2i–k).

### BCG-induced trained immunity correlates with lower parasitemia

**Fig. 3:**
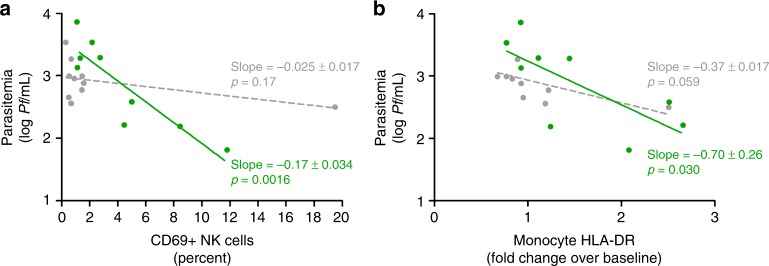
Early NK cell and monocyte activation correlates with decreased parasitemia. Correlations between **a** NK cell CD69 expression and log parasitemia on day 7 after challenge infection, and **b** increase in monocyte HLA-DR MFI and log parasitemia on day 7 are shown for BCG vaccinated (*n* = 9, green) and control (*n* = 10, grey) volunteers. Lines show the result of linear regression analysis for both groups

We next evaluated whether this altered innate immune phenotype had consequences for control of parasitemia. While at group level this primary endpoint was not statistically met, we identify a subgroup of approximately half of BCG vaccinated volunteers with lower levels of parasitemia after challenge. These effects were correlated with changes in the immune parameters defined as secondary endpoints. Indeed, the subset of BCG vaccinated volunteers with early lymphocyte and monocyte activation were also those with lower parasitemia within the BCG group (Fig. 3a, b and Supplementary Fig. 6), and early NK cell CD69 expression and monocyte HLA-DR expression were correlated with decreased parasitemia. In contrast, increased neutrophil activation in BCG vaccinated volunteers was not associated with decreased parasitemia (Supplementary Fig. 7).

### BCG augments *P. falciparum*-induced cytotoxic lymphocyte responses

**Fig. 4:**
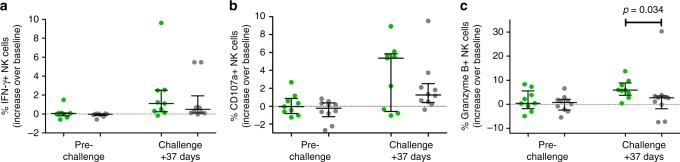
BCG vaccination increases NK cell responses against *P. falciparum*. **a** Percentage of total NK cells staining positive for IFN-γ after 24 h of stimulation with *Pf*RBC before, and 37 days after malaria challenge infection as compared to baseline (pre-BCG vaccination time point), each dot represents an individual BCG vaccinated (*n* = 9, green) or control volunteers (*n* = 10, grey). **b** Percent NK cells staining positive for the degranulation marker CD107a. **c** Percent NK cells staining positive for granzyme B. Lines and error bars show median and interquartile range. *p*-values are the result of Mann–Whitney *U*-test. *p < 0.05. Stimulation was performed in duplo, with replicates combined for flow cytometry analysis

NK cells stimulated with *P. falciparum-*infected red blood cells (*Pf*RBC) in vitro showed no difference in degranulation (defined by CD107a staining), IFN-γ or granzyme B production in BCG vaccinated versus controls prior to challenge infection (Fig. 4). However, 37 days after challenge infection, NK cells from BCG vaccinated volunteers produced significantly more granzyme B (Mann–Whitney *U*-test: *p* = 0.03) with a tendency towards increased degranulation, compared to controls.

The induction of T-cell responses after CHMI was also analysed in both groups (Supplementary Fig. 8). Again, there were no measurable differences between the groups after BCG vaccination and prior to malaria infection. However, post CHMI there were more *P. falciparum*-specific CD4+ T cells producing granzyme B in BCG vaccinated volunteers (Mann–Whitney *U*-test: *p* = 0.02). Furthermore, there were trends towards increased degranulation and granyzme B production in γδT cells.

Finally, the induction of *P. falciparum*-specific antibody responses was analysed in both groups. There were no differences in antibody responses to the immunodominant Circumsporozoite protein (CSP) expressed on sporozoite stages, the liver stage antigen (LSA) expressed on liver stages or total lysate of asexual blood stages (Supplementary Fig. 9a–c). Furthermore, BCG vaccination did not influence the ability of antibodies to block sporozoite invasion into the HC-04 hepatoma cell line (Supplementary Fig. 9d).

## Discussion

Here, we provide in vivo evidence suggestive of the induction of functional trained immunity by BCG vaccination against a heterologous, clinically relevant human pathogen. The existence of trained immunity after BCG vaccination has been previously demonstrated in vitro^4,5,15^ and in murine models^6,8^. However, translation of such findings into equivalent human responses in vivo has so far been limited to a single study where BCG reduced viremia after vaccination with the non-pathogenic, live-attenuated yellow fever vaccine^16^.

The vaccinated volunteers develop early clinical symptoms and laboratory abnormalities, and earlier and stronger inflammatory responses in a subset of volunteers are associated with lower parasitemia. This altered course of immune activation in BCG vaccinated individuals sharply contrasts with CHMIs in control volunteers of this and previous studies, where innate immune activation is only detectable just prior to microscopic parasitemia^17–19^, i.e., at 3−4 days after parasites emerge from the liver. In addition, the course of clinical symptoms is strikingly different from that seen in other, similar CHMI studies at our centre, where symptoms are typically absent on day 7 post-challenge^20–22^. The prompt activation of NK cells in BCG vaccinated volunteers apparently represents a true memory phenotype rather than persistent inflammation, as immediately prior to CHMI there was no difference in activation of peripheral blood lymphocytes between the control and test groups.

Interestingly, the earlier and stronger immune activation markers in half the BCG vaccinated volunteers correlated with a reduced parasitemia in early infection, whereas those with higher parasitemia had no immune activation on day 7 after challenge. This may be due to either reduced release of parasites emerging from the liver or by rapid clearance of blood stage parasites. Indeed, IFN-γ produced by liver lymphocytes in mice suppresses schizont development and subsequent parasitemia^23,24^. In this study we neither detect peripheral blood leucocyte activation, nor increases in circulating IFN-γ or CRP during the liver stage. However, the contribution of undetected local inflammatory processes in the liver cannot be excluded. More sensitive techniques such as (single-cell) transcriptomic analysis may be needed to study peripheral blood responses during the liver stage. Alternatively, the observed reduction of parasite load after BCG vaccination may be the result of efficient asexual clearance as previously shown in C57BL/6 mice infected with *P. yoelii*^12^. In the current CHMI, estimation of asexual parasite multiplication has not been possible since curative treatment was administered at very low parasite densities. In future studies, this might be addressed by allowing longer duration of parasitemia, or alternatively, by using a blood stage challenge infection with a low inoculum, which would allow for even longer exposure to blood stage parasites.

The changes in the clinical and parasitological outcomes in BCG vaccinated volunteers are also associated with *P. falciparum-*specific elicited cellular immune responses after CHMI, including an improved *P. falciparum*-induced NK cell granzyme B production and a trend towards increased degranulation. Such memory-like NK cell responses after CHMI have been described previously^25^, and found to be T-cell dependent. In addition, the number of granzyme B producing CD4+ T cells in response to *P. falciparum* is also increased with a trend towards increased CD4+ T cell IFN-γ production and γδT cell granzyme B production and degranulation. The combined data do suggest that the altered kinetics of immune cell activation in BCG vaccinated volunteers may improve their ability to generate *P. falciparum*-specific responses as has been shown in relation to other vaccines^26^. However, a CHMI with five mosquito bites is not likely to induce significant cellular or humoral immunity, and this hypothesis should be tested in a study combining BCG with a malaria vaccine.

A recent study examined the epigenetic and transcriptomic changes in monocytes of healthy volunteers vaccinated with BCG^16^, showing genome-wide changes in histone H3 acetylation at lysine 27 (H3K27ac) in ‘responding’ volunteers. Our study finds functional changes in NK cells as well, confirming previous in vitro observations^15^. This may be the result of increased monocyte activation, as NK cell activity against malaria is partially dependent on monocytes^27^. Whether BCG induces epigenetic changes in NK cells as well should be subject of a future study.

This study is limited by its small sample size and the subsequent lack of sufficient power for comprehensive statistical analysis of all immune responses. However, the fact that strong correlations can be found in such a small sample size is encouraging. It is striking that there is such clear dichotomy between volunteers, with 4 out of 9 ‘responders’, showing accelerated immune responses and a relative decrease in parasitemia. Interestingly, the ‘non-responders’ in this cohort seem to have increased parasitemia compared to the controls. Other studies with BCG-induced trained innate immunity have also identified significant variability in responses between individuals^28^. The small size of this study, however, does prohibit a clear identification of the factors that predict the effect of BCG vaccination, and future larger cohort studies are needed to explore the factors underlying this variation.

For this study, the observation period of 5 weeks was chosen based on evidence of BCG-induced protection against malaria in mice at 1–2 months post vaccination^9–12^ and BCG-induced trained innate immunity in humans at 2 weeks and 3 months post vaccination^5^. Since the observation period is limited to 5 weeks, it will be important in future studies to determine the duration of this effect, even more so as the in vitro effects seem to persist up to a year after vaccination^4^.

Yet even in the possible absence of longer term effects, these findings may still have clinical implications for malaria as BCG vaccination may improve immunity to malaria, before sufficient adaptive immune responses have been generated to prevent (severe) disease. Although its immune modulatory properties have been known for decades^29^, BCG is currently facing renewed interest after randomised controlled trials have shown it decreases neonatal mortality due to sepsis and respiratory infections^30,31^. There is limited data on BCG and the incidence of malaria from observational studies, with one study showing a reduction in malaria mortality in BCG vaccinated infants^13^. Non-specific beneficial clinical effects of BCG vaccination might be explained by trained innate immunity^32,33^, supporting the further exploration of these effects to better inform the place of BCG in vaccination regimens. Although BCG vaccination is common practice in malaria-endemic countries as part of the WHO Expanded Programme on Immunisation, potential efficacy against malaria and other pathogens underscores the need for investment in timely and correct BCG administration. Epidemiological data and randomised trials suggest revaccination with live-attenuated vaccines such as BCG confers additional protection against all cause mortality^34^. It will be important to determine whether BCG revaccination induces non-specific beneficial effects against malaria. Although BCG revaccination did not reduce malaria morbidity in one study in Guinea-Bissau^35^ potential confounding effects of other vaccines, including DTP with known interference with the overall non-specific effects of BCG^36^ was not taken into account.

In conclusion, BCG vaccination alters some of the the clinical, immunological and parasitological outcomes of malaria infection in a subset of volunteers. Earlier NK cell and monocyte activation in this subset of vaccinated volunteers is consistent with the possibility that induction of trained innate immunity in vivo may have functional activity against a heterologous pathogen in humans. These findings may open perspectives and pathways for clinical vaccine development.

## Methods

### Clinical trial

This single-centre, single-blinded randomised controlled trial was conducted at the Radboud university medical centre (Nijmegen, The Netherlands) from August 2016 until February 2017. Prior to inclusion, study volunteers were medically screened as described previously^37^ and provided written informed consent. The trial was approved by the Central Committee on Research Involving Human Subjects (CCMO NL56222.091.15) of the Netherlands, performed according to the Declaration of Helsinki and Good Clinical Practice and prospectively registered at ClinicalTrials.gov (NCT02692963).

Twenty healthy, BCG-naive volunteers (age 18–35 years) without a history of malaria or residence in a malaria-endemic area in the 6 months before study entry were included and randomly assigned to two groups. Male and female volunteers were allocated separately, to ensure an equal distribution between groups. Volunteers had not received any other vaccinations within 3 months of enrolment. Ten subjects received standard dose (0.1 mL of the reconstituted vaccine) of intradermal BCG vaccination (BCG Bulgaria, Intervax) 5 weeks prior to challenge infection. Ten controls (group 2) received no vaccination.

Five weeks after BCG vaccination, both groups (BCG vaccinated, *n* = 9; 1 excluded after BCG vaccination and controls, *n* = 10) were exposed to bites of five *Plasmodium falciparum* NF54 strain infected *Anopheles stephensi* mosquitoes (sporozoite challenge). Details on the challenge infection are provided in Supplementary Table 3. Subjects and investigators were not blinded, whereas those performing the qPCR analysis were blinded until after the last qPCR data had been collected. qPCR was performed prospectively, once daily from day 6 after CHMI until day 3 after antimalarial treatment, according to previously published protocols^22,38,39^. All volunteers were treated with a curative regimen of antimalarial drugs (atovaquone/proguanil) once the treatment threshold of 100 parasites/mL blood was exceeded detected by qPCR or presumptively on day 21 after challenge if qPCR remained below treatment threshold.

### Recording of adverse events

Subjects recorded clinical symptoms in a diary, from the time of BCG vaccination until 37 days after the CHMI. Both solicited and unsolicited adverse events were recorded after questioning by the investigators at set time points: prior to BCG vaccination, prior to the CHMI, daily from day 6 after infection until 3 days after antimalarial treatment, and on day 37 post CHMI^21,40^. Adverse events were graded according to criteria defined in the Clinical Trial Protocol: mild (grade 1): awareness of symptoms that are easily tolerated and do not interfere with usual daily activity; moderate (grade 2): discomfort that interferes with or limits usual daily activity; severe (grade 3): disabling, with subsequent inability to perform usual daily activity, resulting in absence or required bed rest. Relatedness was assessed by the investigator, also on the bases of pre-defined criteria: probable: an adverse event that follows a reasonable temporal sequence from the challenge procedure and cannot be reasonably explained by the known characteristics of the subject’s clinical state; possible: an adverse event for which insufficient information exists to exclude that the event is related to the study procedure; not related: an event for which sufficient information exists to indicate that the aetiology is unrelated either because of the temporal sequence of events or because of the subject’s clinical state or other therapies.

Oral temperature was measured by volunteers and recorded in the symptom diary every morning and more frequently during symptoms. Tympanic temperature was measured by the study physician at every follow-up visit. Fever was scored as follows: mild (grade 1): 37.6–38.0 °C; moderate (grade 2): 38.1–39.0 °C; severe (grade 3): ≥39.1 °C.

### Whole blood flow cytometry

One-hundred microlitres(lymphocytes) or 50 µL (monocytes and neutrophils) of fresh EDTA blood was stained directly with antibodies. For lymphocyte analysis, samples were stained with CD3-AlexaFluor700 (Biolegend; clone OKT3; catalogue number 317340; final dilution 1:640), pan-γδTCR^−^PE (Beckman Coulter; clone IMMU510; catalogue number COIM1349; final dilution 1:160), CD56-Brilliant Violet(BV)421 (Biolegend; clone HCD56; catalogue number 318328; final dilution 1:320), CD16-APC-eFluor780 (eBiosciences; clone CB16; catalogue number 47–0168–42; final dilution 1:640), CD69-PerCP-Cy5.5 (Biolegend; clone FN50; catalogue number 310926; final dilution 1:640). For monocyte analysis, samples were stained with a lineage mix containing CD3-PerCP-Cy5.5 (Biolegend; clone HIT3a; catalogue number 300328; final dilution 1:400), CD19-PerCP-Cy5.5 (Biolegend; clone HIB19; catalogue number 302230; final dilution 1:200) and CD56-PerCP-Cy5.5 (Biolegend; clone HCD56; catalogue number 318322; final dilution 1:100), CD14-FITC (Biolegend; clone HCD14; catalogue number 325604; final dilution 1:80), CD16-PE-Cy7 (Biolegend; clone 3G8; catalogue number 302016; final dilution 1:1280), HLA-DR−APC-Cy7 (Biolegend; clone L243; catalogue number 307618; final dilution 1:160) and CD86−Pacific Blue (Biolegend; clone IT2.2; catalogue number 305423; final dilution 1:100). For neutrophil analysis samples were stained with CD14-PerCP (Biolegend; clone HCD14; catalogue number 325632; final dilution 1:30), HLA-DR-APC (Biolegend; clone L243; catalogue number 307610; final dilution 1:80), CD16^−^APC-eFluor780 (eBiosciences; clone CB16; catalogue number 47–0168–42; final dilution 1:1280), CD62L-PE-Cy7 (eBioscience; clone DREG-56; catalogue number 25–0629–42; final dilution 1:1280) and CD11b-BV510 (Biolegend; clone ICRF44; catalogue number 301334; final dilution 1:180). Samples were stained for 30 min at 4° C (C) in the dark. After staining, erythrocytes were lysed for 5 min at 4 °C with 1 mL BD FACS Lysis buffer, followed by centrifugation. Cell pellets were washed once with 1 mL FACS buffer (0.5% bovine serum albumin (BSA) in PBS) and resuspended in PBS with 1% paraformaldehyde (PFA) and analysed on a Gallios flow cytometer (Beckman Coulter) the same day. Flow cytometry data was analysed using Flow Jo software (version 10.0.8 for Apple OS). The gating strategy and representative plots are shown in Supplementary Fig. 10.

### PBMC isolation, cryopreservation and thawing

Blood samples for peripheral blood mononuclear cell (PBMC) isolation were collected at inclusion (incl), prior to challenge (C−1) and 37 and 121 days after challenge infection (C + 37, C + 121). PBMC were isolated by density gradient centrifugation from citrate anti-coagulated blood using vacutainer cell preparation tubes (CPT; BD Diagnostics). Following four washes in ice-cold phosphate buffered saline (PBS), cells were counted and cryopreserved at a concentration of 10 × 10^6 ^cells/mL in ice-cold foetal calf serum (Gibco)/10% DMSO (Merck) using Mr. Frosty freezing containers (Nalgene). Samples were stored in vapour-phase nitrogen. Immediately prior to use, cells were thawed, washed twice in Dutch-modified RPMI 1640 (Gibco/Invitrogen) and counted in 0·1% Trypan blue with 5% Zap-o-Globin II Lytic Reagent (Beckman Coulter) to assess cell viability.

### PBMC restimulation

For lymphocyte responses, PBMCs taken at inclusion, C-1, C + 37, and C + 121 were stimulated with purified NF54 strain schizonts or uninfected erythrocytes. Cells were cultured in RPMI 1640 (Dutch Modification; Gibco) with 5 mg/mL gentamycin (Centraform), 100 mM pyruvate (Gibco), 200 mM glutamax (Gibco), supplemented with 10% heat-inactivated pooled human A + serum (obtained from Sanquin Bloodbank, Nijmegen, The Netherlands). Anti-CD107a-Pacific Blue antibody (Biolegend; clone H4A3; catalogue number 328624; final dilution 1:400) was added throughout co-culture. Brefeldin A (10 µg/mL; Sigma-Aldrich) and monansin (2 µM; eBioscience) were added after 20 h. After an additional 4 h of stimulation, cells were washed and stained with a fixable viability dye labelled with eFlour780 (eBioscience) for 30 min at 4° C. After washing cells were stained with antibodies against surface markers: CD3-ECD (Beckman Coulter; clone UCHT1; catalogue number A07748; final dilution 1:100), CD4-FITC (BD Biosciences; clone SK3; catalogue number 340133; final dilution 1:20), CD8-AlexaFluor700 (Biolegend; clone HIT8A; catalogue number 300920; final dilution 1:2000), pan-γδTCR-PE (Beckman Coulter; clone IMMU510; catalogue number COIM1349; final dilution 1:160), and CD56-PerCP-Cy5.5 (Biolegend; clone HCD56; catalogue number 318322; final dilution 1:100), for 30 min at 4 degrees. Cells were washed and fixed with Foxp3 fixation/permeabilization buffer (eBioscience) for 30 min at 4 degrees. After washing with permeabilization buffer (eBioscience) cells were stained for intracellular cytokines with IFN-γ-PE-Cy7 (Biolegend; clone 4 S.B3; catalogue number 502528; final dilution 1:200) and granzyme B-AlexaFluor647 (Biolegend; clone GB11; catalogue number 515406; final dilution 1:200) for 30 min at 4 degrees. After washing with permeabilization buffer, cells were taken up in PBS with 1% PFA. Cells stimulated PMA (10 ng/mL; Sigma) and ionomycin (1 µg/mL; Sigma) for 4 h were used as a positive control.

Samples were analysed on a Gallios flow cytometer (Beckman Coulter) the same day. Flow cytometry data was analysed using Flow Jo software (version 10.0.8 for Apple OS). CD107a and cytokine responses to PfRBC were corrected for uRBC at every time point (thus, defined as percent increase over background), and then corrected for baseline (pre-vaccination) responses. Gating strategy and representative plots are shown in Supplementary Fig. 11.

### Circulating cytokines and granzyme B

Plasma concentrations of TNF-α, IL-1β, (detection range 0.98–4000 pg/mL) IL-6 (0.36−1500 pg/mL), IL-8 (0.62–2500 pg/mL), IL-10, (2.92–12,000 pg/mL) IFN-γ (1.22–5000 pg/mL) and granzyme B (2–10.000 pg/mL) were measured in citrate plasma using a Luminex assay according to the manufacturer’s instructions (Milliplex, Merck Millipore, Billerica, MA, USA).

### High sensitivity C-reactive protein

Automated hsCRP measurements were performed on citrated plasma samples with immunonephelometry with a Behring Nephelometer Analyser following the manufacturers’ instructions, using reagents and calibrators specifically designed for high sensitivity measurements. The detection limit was 0.16 mg/L.

### Malaria-specific antibody ELISA

Malaria-specific antibody levels were determined by standardised ELISA as described previously^41^. In short, plates were coated with circumsporozoite protein (CSP), liver stage antigen-1 (LSA1) protein or lysed ring stage parasites. Citrated plasma from volunteers was diluted 50x and 150x and analysed in duplicate. A standard curve was generated by serial twofold dilutions of serum from a pool of 100 Tanzanian adults living in an endemic area (HIT serum). ELISA data analysis was performed with Auditable Data Analysis and Management System for ELISA (ADAMSEL, version 1.1). Post-challenge plasma samples were corrected for pre-challenge responses.

### Sporozoite invasion assay

HC-04 human hepatoma cells (obtained from MR4) were seeded in collagen coated 96-well plates (coated with 0.056 mg/mL for 1 h; Collagen from Rat Tail, Sigma-Aldrich) at 50,000 cells per well. Sixteen hours after seeding, NF54 *P. falciparum* sporozoites were pre-incubated on ice for 30 min with 10% heat-inactivated pre- or post-challenge citrate plasma from volunteers and 10% heat-inactivated serum from non-immune adult. Sporozoites incubated with 10% heat-inactivated serum from highly immune Tanzanian adults and 10% non-immune serum, or 20% non-immune serum served as positive and negative control, respectively. Following pre-incubation, 50,000 sporozoites were added per well in triplicate. Plates were centrifuged at 3000 rpm for 10 min (Eppendorf Centrifuge 5810R) and incubated for 3 h on 37 °C, 5% CO_2_.

After three hours, wells were washed three times with PBS to remove medium, antibodies and non-invaded sporozoites. Subsequently, cells and any extracellular adherent sporozoites were dissociated by incubating with 0.05% trypsin with EDTA (ThermoFisher) for 5 min at 37 °C, followed by neutralisation with an equal volume 10% heat-inactivated human serum in PBS. Cells were transferred into 96-well V-bottom plates, spun down at 1700 rpm for 4 min at 4 °C.

Cells were washed with PBS and fixed with Foxp3 fixation/permeabilization buffer (eBioscience). After washing with permeabilization buffer (eBioscience), intracellular sporozoites were stained with FITC-labelled 3SP2 antibody (monoclonal antibody against CSP, published previously^42^) for 30 min at 4 °C. After washing in permeabilization buffer cells were taken up in 1% paraformaldehyde and analysed on a Gallios flow cytometer (Beckman Coulter) the same day.

Flow cytometry data was analysed using Flow Jo software (version 10.0.8 for Apple OS). Live cells were gated based on forward scatter/sideways scatter characteristics and percent invasion was defined as percentage of live cells positive for FITC. Post-challenge samples were compared to pre-challenge samples.

## Supplementary information

Peer Review File

Supplementary Information

## Data Availability

The datasets used and/or analysed during the current study are available from the corresponding author upon request. A reporting summary for this Article is available as a Supplementary Information file.
