# Supplementary material for: Outcomes of controlled human malaria infection after BCG vaccination
Source: Nat Commun. 2019 Feb 20;10:874. doi: 10.1038/s41467-019-08659-3 (PMC6382772; doi:10.1038/s41467-019-08659-3)

Outcomes of Controlled Human Malaria Infection after BCG vaccination

Walk and de Bree et al.

Supplementary Data

Supplementary figure 1: clinical trial flow chart. BMI = Body Mass Index; EBV = Epstein-Barr Virus

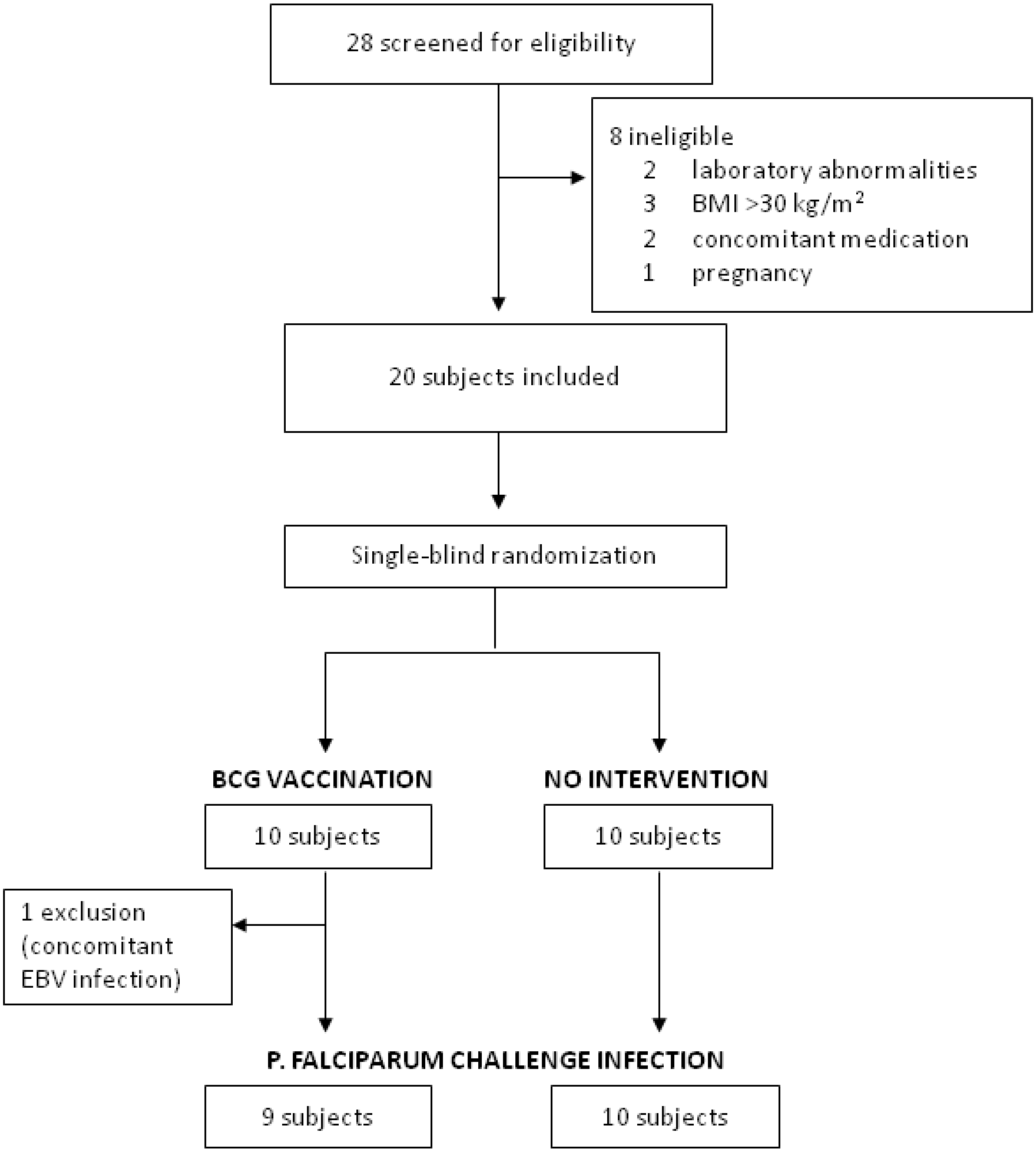

Supplementary table 1: baseline characteristics of study subjects.

	BCG vaccinated (n=10)	Controls (n=10)	Total (n=20)
**Age (years) ± SD**	**21.2 ± 2.1**	**23.0 ± 3.2**	**22.1 ± 2.8**
**Sex (*n* (%))**			
**Female**	7 (70)	6 (60)	13 (65)
**Male**	3 (30)	4 (40)	7 (35)
**Race (*n* (%))**			
**Caucasian**	10 (100)	9 (90)	19 (95)
**Afro-Caribbean**	0 (0)	1 (10)	1 (5)

Supplementary table 2: adverse events reported by BCG vaccinated and control volunteers. Solicited adverse events were collected from day 6 after the malaria infection until day 3 after antimalarial treatment. Below the number and percent of BCG vaccinated or control volunteers reporting a specific adverse event is listed, for adverse events occurring before antimalarial treatment and adverse events reported after antimalarial treatment.

	Pre-treatment		Post-treatment	
	BCG	Control	BCG	Control
**Headache**	**5 (56%)**	**2 (20%)**	**8 (89%)**	**4 (40%)**
grade 1	4 (44%)	2 (20%)	5 (56%)	2 (20%)
grade 2	1 (11%)	0 (0%)	3 (33%)	2 (20%)
grade 3	0 (0%)	0 (0%)	0 (0%)	0 (0%)
**Malaise/chills**	**1 (11%)**	**0 (0%)**	**1 (11%)**	**1 (10%)**
grade 1	0 (0%)	0 (0%)	0 (0%)	1 (10%)
grade 2	1 (11%)	0 (0%)	1 (11%)	0 (0%)
grade 3	0 (0%)	0 (0%)	0 (0%)	0 (0%)
**Fever**	**3 (33%)**	**1 (10%)**	**5 (56%)**	**3 (30%)**
grade 1	2 (22%)	0 (0%)	3 (33%)	2 (20%)
grade 2	1 (11%)	1 (10%)	2 (22%)	0 (0%)
grade 3	0 (0%)	0 (0%)	0 (0%)	1 (10%)
**Myalgia/arthalgia**	**2 (22%)**	**1 (10%)**	**3 (33%)**	**3 (30%)**
grade 1	2 (22%)	1 (10%)	2 (22%)	3 (30%)
grade 2	0 (0%)	0 (0%)	1 (11%)	0 (0%)
grade 3	0 (0%)	0 (0%)	0 (0%)	0 (0%)
**Nausea/vomiting**	**4 (44%)**	**0 (0%)**	**5 (56%)**	**1 (10%)**
grade 1	1 (11%)	0 (0%)	3 (33%)	0 (0%)
grade 2	0 (0%)	0 (0%)	1 (11%)	0 (0%)
grade 3	3 (33%)	0 (0%)	1 (11%)	1 (10%)
**Abdominal pain**	**0 (0%)**	**1 (10%)**	**1 (11%)**	**1 (10%)**
grade 1	0 (0%)	1 (10%)	1 (11%)	0 (0%)
grade 2	0 (0%)	0 (0%)	0 (0%)	1 (10%)
grade 3	0 (0%)	0 (0%)	0 (0%)	0 (0%)
**Diarrhoea**	**1 (11%)**	**0 (0%)**	**3 (33%)**	**1 (10%)**
grade 1	1 (11%)	0 (0%)	3 (33%)	1 (10%)
grade 2	0 (0%)	0 (0%)	0 (0%)	0 (0%)
grade 3	0 (0%)	0 (0%)	0 (0%)	0 (0%)
**Fatigue**	**3 (33%)**	**1 (10%)**	**5 (56%)**	**0 (0%)**
grade 1	1 (11%)	0 (0%)	4 (44%)	0 (0%)
grade 2	1 (11%)	1 (10%)	1 (11%)	0 (0%)
grade 3	1 (11%)	0 (0%)	0 (0%)	0 (0%)

Supplementary figure 2: clinical symptoms in BCG and control volunteers versus historical controls. (A) Adverse events were collected daily. The Kaplan-Meier curve shows the percentage of volunteers experiencing one or more moderate or severe, solicited, symptoms during follow-up on day 5-7 after challenge and day 1-3 after antimalarial treatment. BCG vaccinated volunteers (green) experienced earlier and more moderate/severe symptoms than controls (blue). 35 volunteers participating in other CHMI studies using the same parasite strain and the same treatment criteria (grey) also differed significantly from the BCG vaccinated volunteers but not the controls.

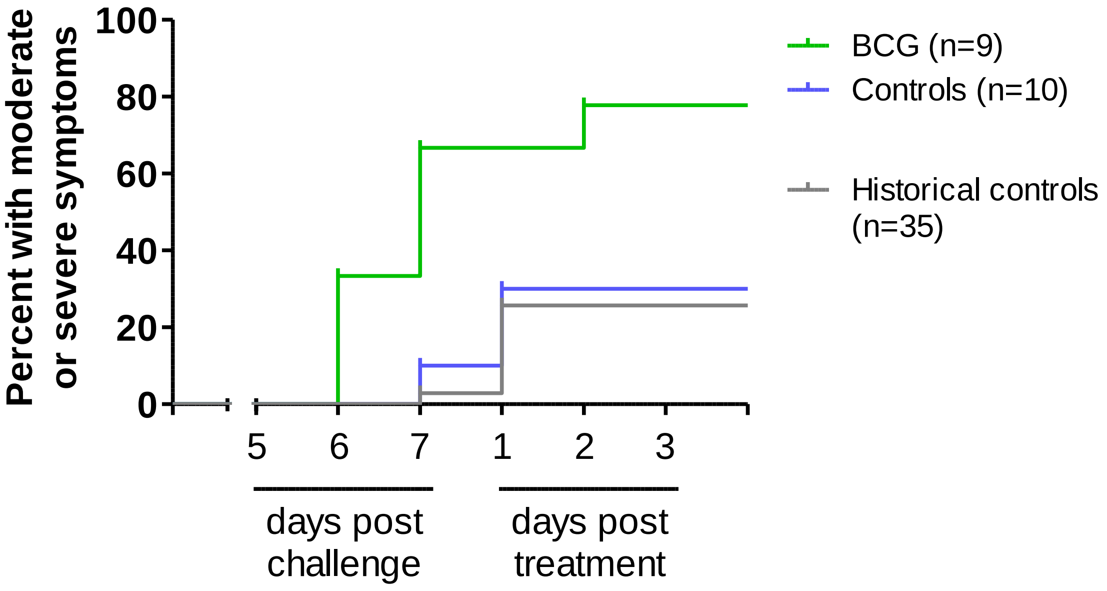

Log-rank (Mantel-Cox) Test

BCG vs. Controls	BCG vs. Historical	Controls vs. Historical
**p=0.014**	**p<0.0001**	**p=0.72**

Supplementary figure 3: Platelet decreases during infection in BCG and control volunteers. Graph shows the relative change in circulating platelets between baseline and the lowest measurement during follow-up in BCG vaccinated (green) versus control (grey) volunteers. P-value is the result of a student's t-test.

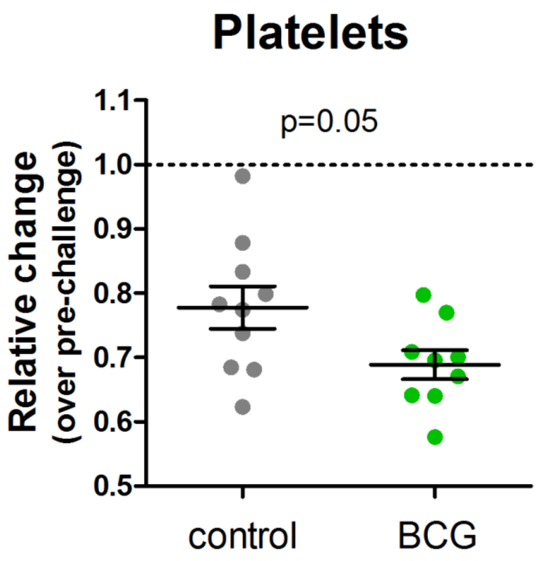

Supplementary figure 4: temperature in BCG vaccinated and control volunteers. Tympanic temperature was measured daily and at moment of antimalarial treatment in BCG vaccinated (green) and control (blue) volunteers. The graph shows temperature on day 6 and 7 post infection, at treatment and on day 1-3 post treatment for all volunteers.

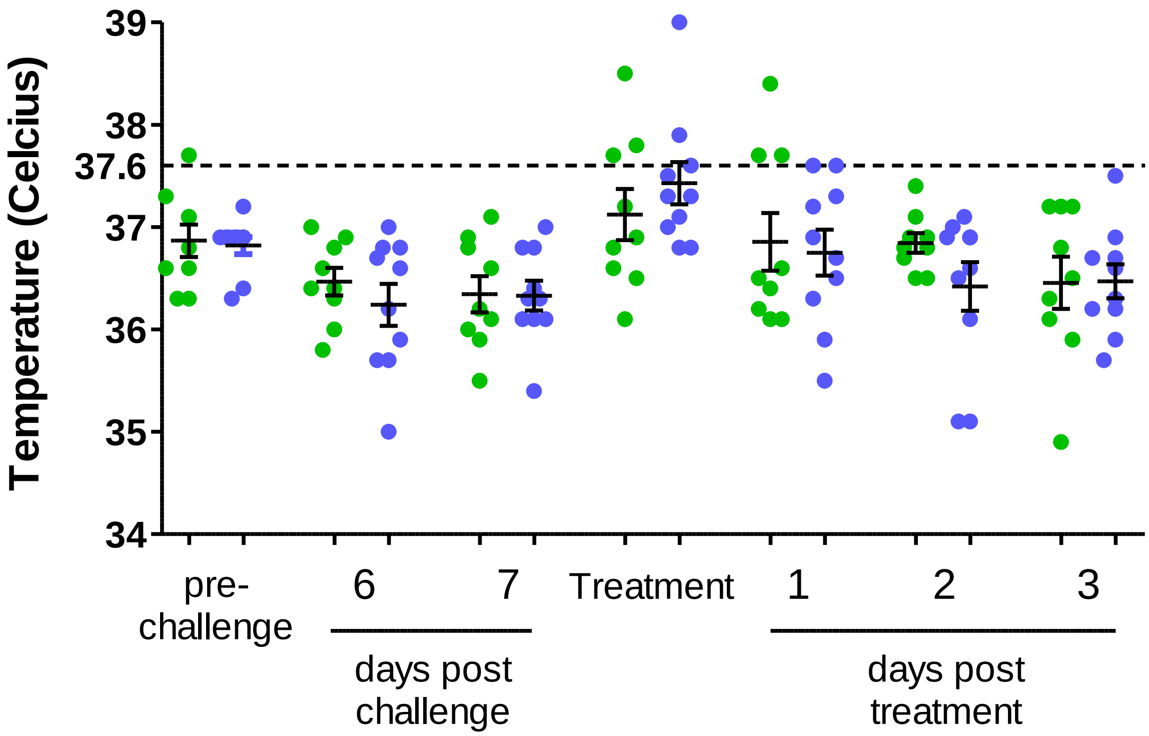

Supplementary figure 5: *in vivo* activation of CD56^{bright}CD16- NK cells. *In vivo* leukocyte activation was determined by direct staining of fresh whole blood with fluorescent antibodies every two days post challenge. Lymphocytes were defined based on forward scatter and sideward scatter characteristics, and duplet events were excluded. NK cell activation was defined as the percentage of CD3$^-$ CD56^{bright}CD16$^-$ live cells expressing CD69. The grey dots show the non-BCG vaccinated control group volunteers (n=10) and each colored dot shows an individual BCG vaccinated volunteer (n=9). Lines and error bars show the median and interquartile range of each group.

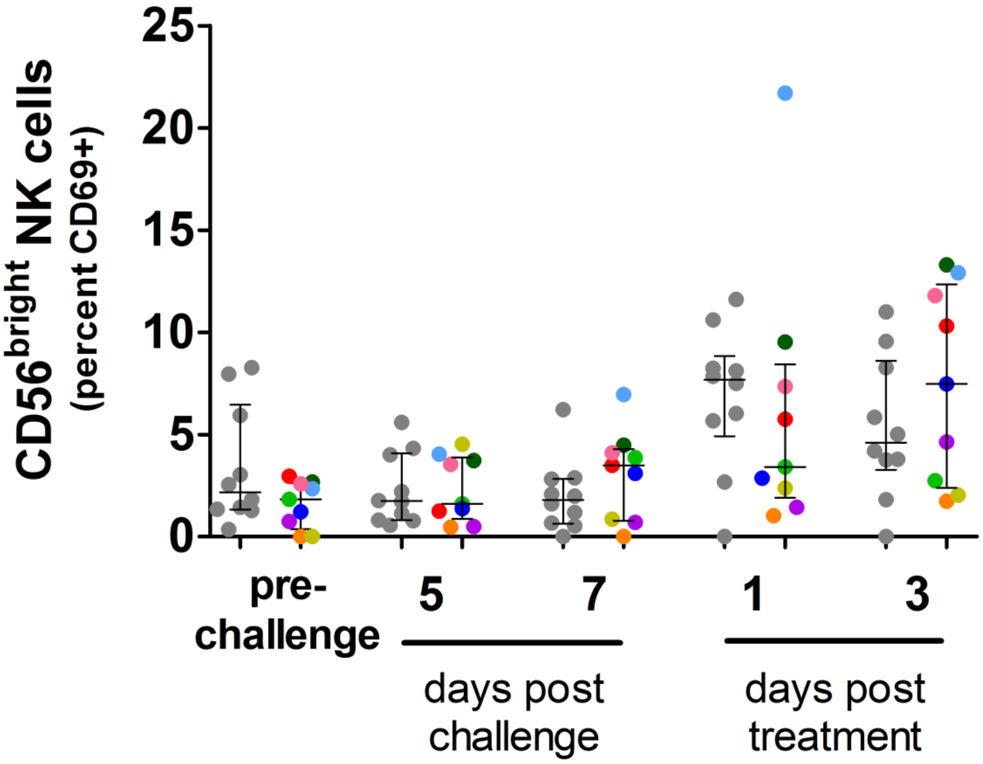

Supplementary figure 6: BCG vaccinated volunteers with accelerated immune activation had lower parasitemia. (A) Correlations between CD69 expression in different lymphocyte subset and log parasitemia on day 7 after challenge infection, (B) percentage $CD16^+$ monocytes and increase in $CD16^-$ monocyte CD86 MFI and log parasitemia on day 7 are shown for BCG vaccinated volunteers (green) and control volunteers (grey). Lines show the result of linear regression analysis, p-values are shown if p<0.05.

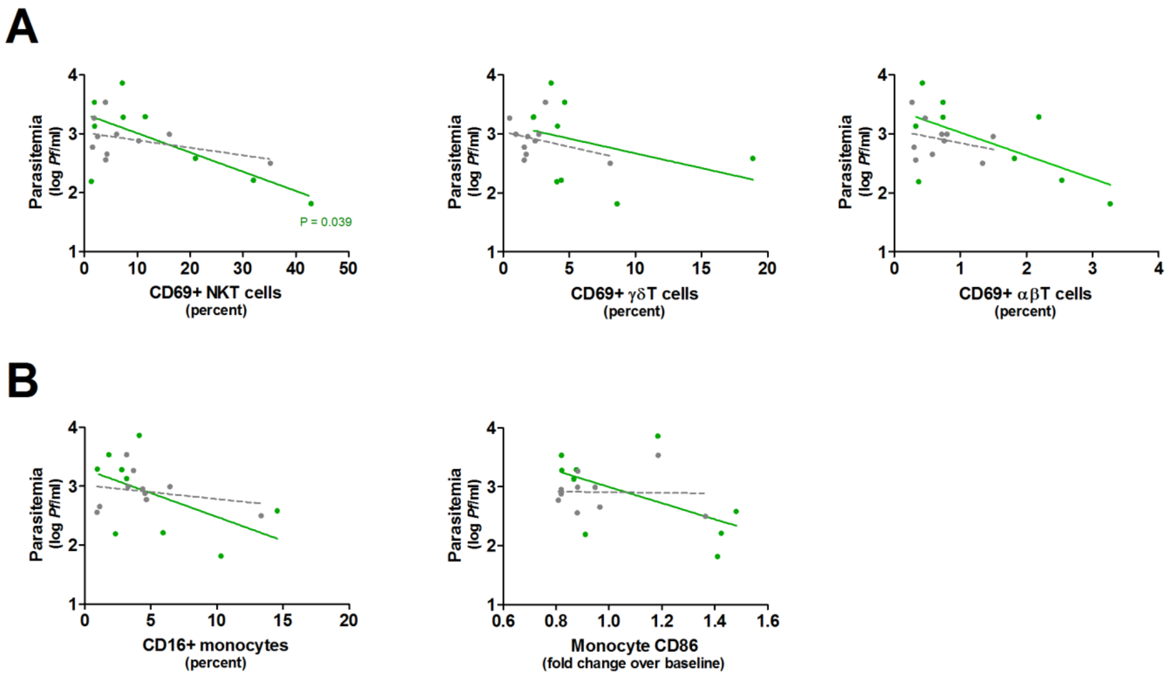

Supplementary figure 7: Neutrophil activation and parasitemia in BCG vaccinated volunteers. Correlations between neutrophil activation, defined as percentage of neutrophils that are CD62L-CD11b^{hi}, and log parasitemia on day 7 after challenge infection are shown for BCG vaccinated (n=9, green) and control (n=10, grey) volunteers. Lines show the result of linear regression analysis for both groups.

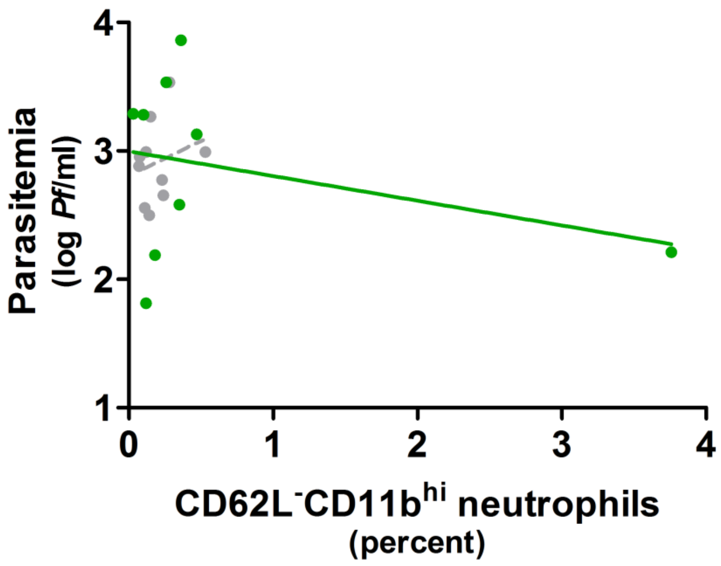

Supplementary figure 8: ***P. falciparum*****-specific T cell responses in BCG and control volunteers.** Percentage of cells staining positive for **(A)** CD107a, **(B)** granzyme B and **(C)** IFN-γ after 24 hours of stimulation with *Pf*RBC before, and 37 and 121 days after malaria challenge infection as compared to baseline (pre-BCG vaccination time point) in BCG vaccinated (green) and control volunteers (grey). Lines and error bars show median and interquartile range. P-values are the result of Mann-Whitney U test.

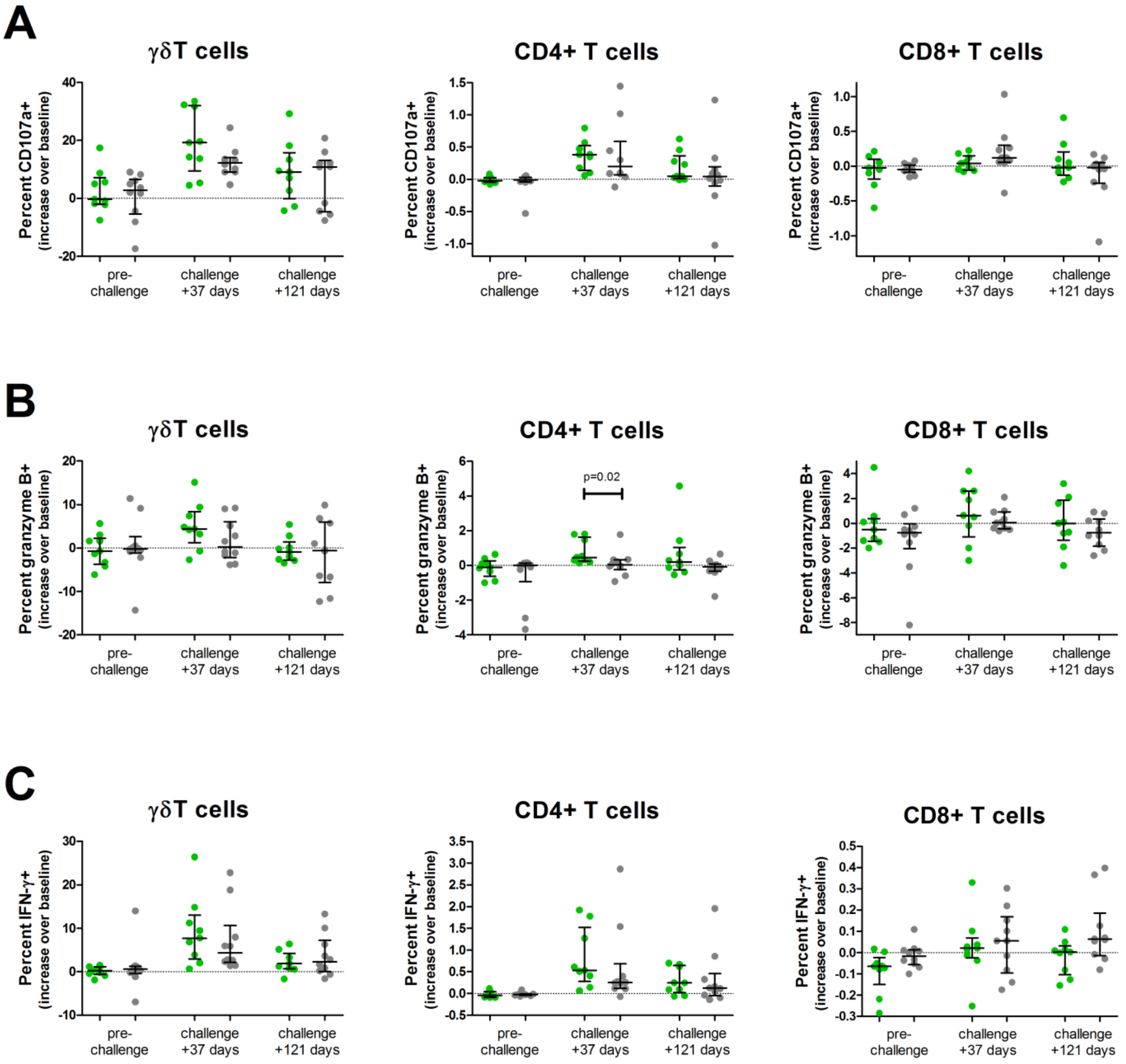

Supplementary figure 9: BCG vaccination does not affect *Pf*-specific antibodies. (A) Antibody titers to circumsporozoite protein (CSP), **(B)** liver stage antigen (LSA) and **(C)** total asexual parasite lysate 37 and 121 days post challenge in BCG vaccinated versus controls. Values are corrected for pre-CHMI levels and shown as percentage of positive control (serum from Tanzanian adults in a highly endemic area). **(D)** Percentage inhibition of sporozoite invasion in HC04 hepatoma cells by plasma taken 37 days post CHMI, controlled for pre-CHMI plasma in BCG vaccinated versus controls.

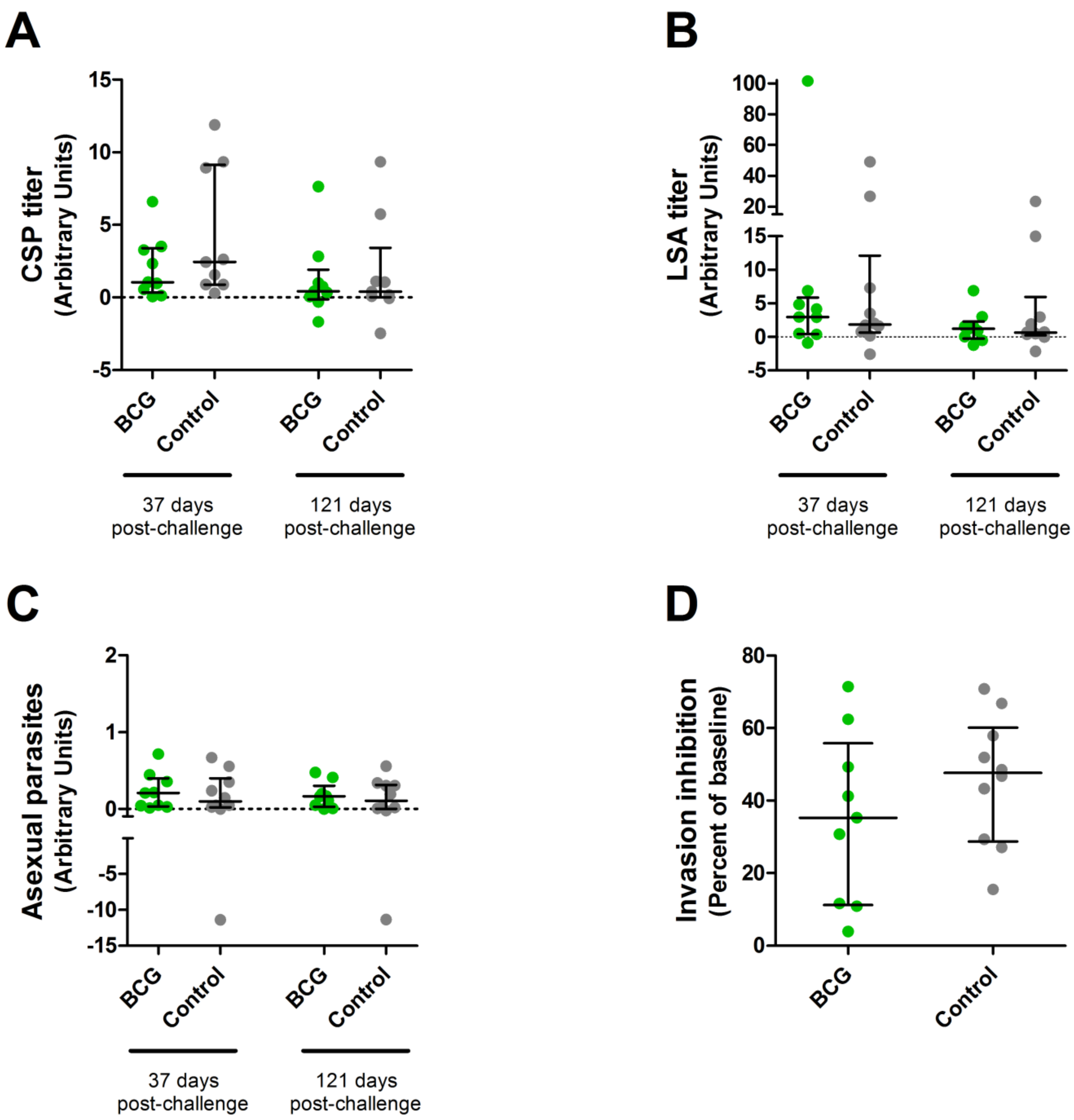

Supplementary table 3: **data on mosquito infection and controlled human malaria infection.** All volunteers were infected with the same batch of *P. falciparum* infected *Anopheles stephansi* mosquitoes. Batch infectivity and mean sporozoite load was determined by dissection of a sample of 10 mosquitoes one day before the challenge infection. All volunteers received exactly 5 bites from infected mosquitoes. Most volunteers required only one or two sessions for a sufficient number of infected mosquito bites, with a single exception who required a third session.

	**Mosquito infectivity**		**Infection**		
	Percent	# Sporozoites per mosquito	Number of sessions median (range)	# Infected bites median (range)	# Uninfected bites median (range)
BCG group	100%	160,500	1 (1-3)	5	0 (0-1)
Control group			1 (1-2)	5	0 (0-1)

Supplementary figure 10: gating strategy for whole blood flow cytometry of lymphocytes (A), monocytes (B) and neutrophils (C).

A

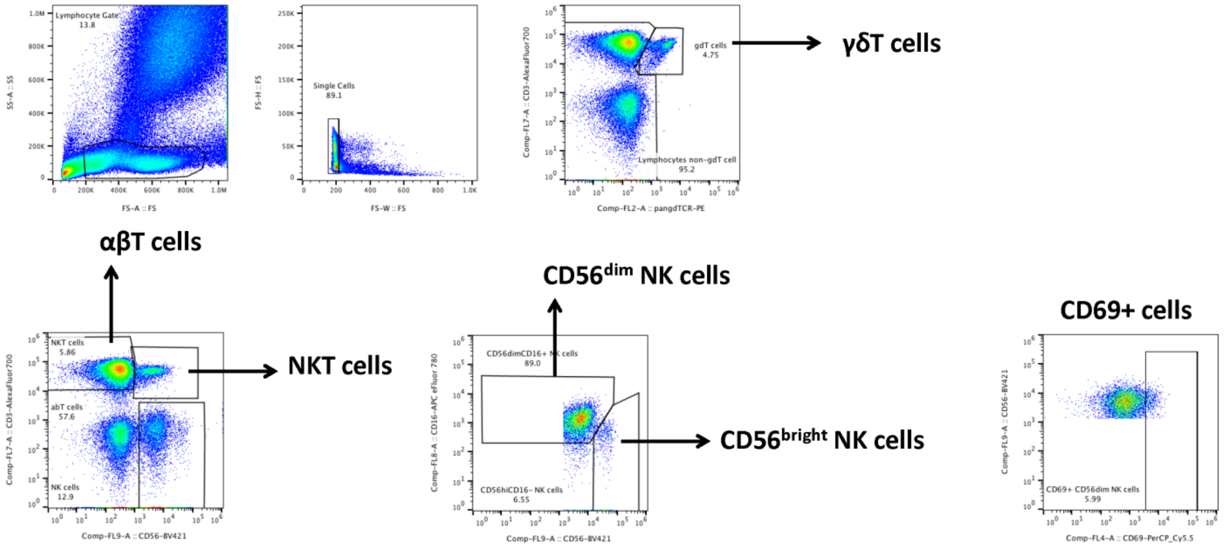

B

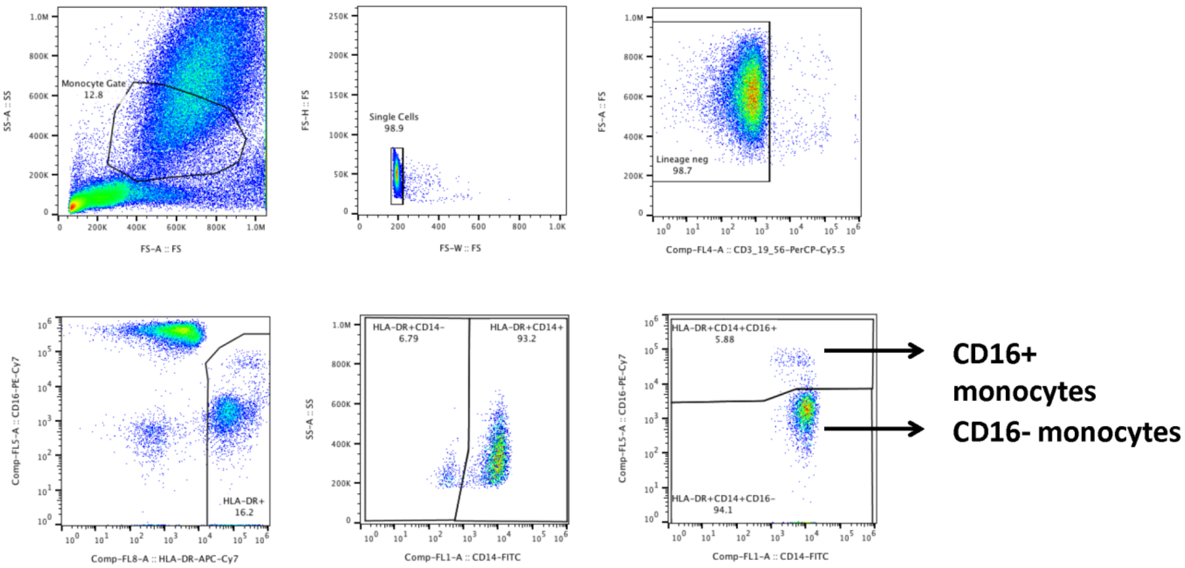

C

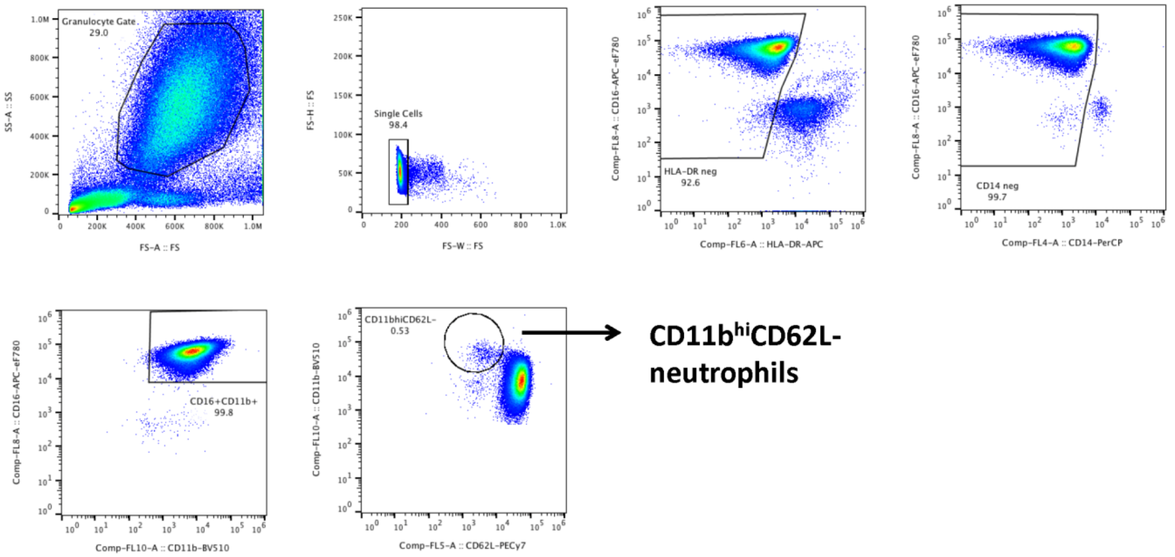
Granulocyte Gate
29.0
SS-A :: SS
FS-A :: FS
Single Cells
98.4
FS-H :: FS
FS-W :: FS
HLA-DR neg
92.6
Comp-FL8-A :: CD16-APC-eF780
Comp-FL6-A :: HLA-DR-APC
CD14 neg
99.7
Comp-FL4-A :: CD14-PerCP
CD16+CD11b+
99.8
Comp-FL10-A :: CD11b-BV510
CD11bhiCD62L-
0.53
Comp-FL10-A :: CD11b-BV510
Comp-FL5-A :: CD62L-PECy7
CD11b^hi CD62L-
neutrophils

Supplementary figure 11: gating strategy *ex vivo P. falciparum* lymphocyte stimulation.

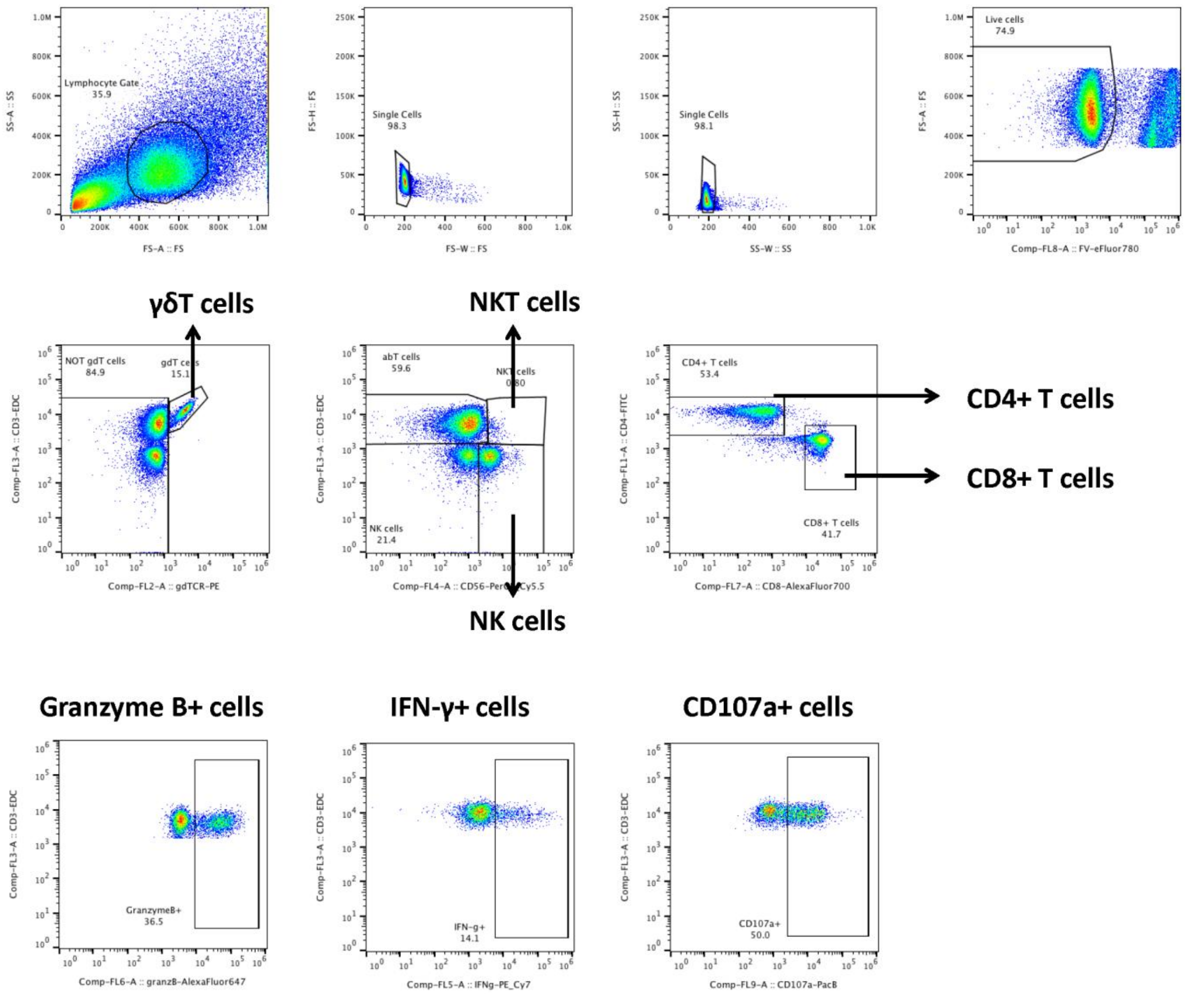

Supplement: Supplementary file 2 — Supplementary Information [file 41467_2019_8659_MOESM2_ESM.pdf]
