# Supplementary material for: Outcomes of controlled human malaria infection after BCG vaccination
Source: Nat Commun. 2019 Feb 20;10:874. doi: 10.1038/s41467-019-08659-3 (PMC6382772; doi:10.1038/s41467-019-08659-3)
Supplement: Supplementary file 1 — Peer Review File [file 41467_2019_8659_MOESM1_ESM.pdf]

Reviewers' comments:

Reviewer #1 (Remarks to the Author):

The authors show that BCG vaccination 5 weeks before controlled human malaria infection results in better control of parasitaemia and increased activation of NK cells and monocytes coinciding with the appearance of blood stage parasites in peripheral circulation. Due to the nature of the experiments, the number of volunteers are small and the changes are observed only in approximately half of the BCG-immunised volunteers but nevertheless significant. The increases in NK and monocyte activation appear to be due to a heightened reactivity of these cells rather than persistent activation and as such form part of the recently described innate or trained memory. The results are interesting and novel in that the effect of BCG vaccination on human malaria have not been investigated previously. However, the mechanisms underlying the increased responsiveness of NK cells after BCG vaccination are not (yet) known or why they occur only in a proportion of BCG vaccinated volunteers. Furthermore, given that BCG vaccination in infancy is part of the EPI programme in Sub-Saharan Africa, benefits for immunity to malaria or changes in vaccination regime to deploy such benefits seem unlikely.

Minor comments

Figure 1 and 2: The same graphs showing changes in platelet, lymphocyte and neutrophil counts (fig 1) as well as changes in leukocyte activation and cytokine levels (fig 2) should be shown for non-BCG vaccinated volunteers.

The authors state in the discussion that BCG vaccination may have implication for improving immunity to malaria but do not further elucidate how that can be achieved. To my knowledge - almost all infants born in sub-Saharan Africa will be immunised with BCG at or shortly after birth and any beneficial effects on severe malarial disease at least during infancy - if the benefits of BCG vaccination last long enough as the authors seem to think - should be present already. Maybe the authors could expand how they see BCG vaccination deployed?

Reviewer #2 (Remarks to the Author):

This manuscript describes a human malaria CHMI study using the standard 5 mosquito-bite model to induce falciparum malaria. This model is usually used to assess the efficacy of vaccines against malaria, however in this study the investigators have assessed the effect of BCG vaccination on immunity to malaria. The rationale for this innovative study is well-described in the introduction and is to assess the impact of inducing innate immunity on protection from malaria.

Unfortunately, while the rationale for the study is well-presented, the results are not and is most cases are either non-significant or only marginally significant. In addition, many of the claims made, particularly in the abstract are not supported by the data presented. Firstly, the authors claim that the BGC-vaccinated participants experienced earlier and more severe clinical symptoms to malaria CHMI, but absolutely no detail is provided as to what these symptoms were or how they were graded and whether these symptoms were reported by participants themselves or observed by investigators (or whether either participants or investigators were blinded). As a consequence, one cannot determine the importance of this observation. Secondly, they claim that these symptoms were associated with the induction of the immune responses that they describe in the BCG-vaccinated group, but no evidence to support this association is presented and this association might be coincidental, particularly given the small numbers in this study. Thirdly, the author suggest that both the enhanced symptoms and induced immunity are associated with reduced parasitaemia at 5 weeks post CHMI, but again there is absolutely no analysis presented to support this. I couldn't even find the PCR data at 5 weeks in the manuscript.

Overall, the findings are weak and add little to the field. The trial is so poorly described that it is hard to determine the importance of the findings in the context presented. If the paper were re-written to describe the study to the standard usually expected, the findings would still be too marginal to be of importance. Most of the immunology figures only show the data for the BCG group, although the text describes comparisons with the unvaccinated group. Both ought to be shown to allow the reader to assess the validity of the claims made.

The study is also largely unreproducible as reported, particularly as the primary endpoint is not described nor the method used to assess it. No information is given about the method for the primary endpoint of the trial (malaria qPCR) and whether the interpretation of PCR data was made by investigators blinded to the vaccination status of the volunteers. It's not stated how often or for how long PCR was performed. One assumes that PCR was performed in real-time, but again this is not described. There is no CONSORT checklist and the T cell methods do not meet the MIATA guidelines, particularly for the ICS itself where the method is minimally described despite ample space in the supplementary to do this properly. The BCG dose is not specified.

The statistical analyses presented are appropriate, although often described in the text and not on the figure itself. Also, it would be useful to know where if a statistic is not given whether a test was performed and a non-significant result obtained and whether correction for multiple analyses were made. A Kaplan-Meier analysis should be performed for the qPCR data during follow-up.

Reviewer #3 (Remarks to the Author):

This is a very interesting study about trained innate immunity in a malaria CHIM, and the authors make a fair case that this can exist. I am generally favorable to the report, although I think that there were some fundamental flaws in study design that cannot be corrected given the effort that is required for CHIM to be undertaken. First and foremost was the relatively short interval between BCG vaccine and challenge. The clinical observations surrounding trained innate immunity occur on a much longer time scale, so 5 weeks seems like a very short time interval between vaccine and challenge. My second objection to the trial design was the very very short period when parasitemia was tolerated. As parasitemia was always sub patent, there was very little to suggest that at least one more day of clinical illness was dangerous to the volunteers. I realize, of course, that Sauerwein and colleagues were probably told by their institutional ethics panel how much clinical illness was acceptable, but this decision contributes to what appear to be significant but small effects that run through the manuscript. A third deficiency, and one that may actually be addressable even at this time point, is the lack of transcriptomic data as well as ChIP seq data. Such data, while expensive and (in the case of ChIP seq) labor intensive, would greatly increase the impact of the manuscript.

All of this being said, this is a piece of work that has importance. Some minor comments: 1) why do the authors believe that BCG vaccinate patients were more symptomatic? This seems like a fairly robust observation, but the numbers are small and the explanation is not obvious. 2) during the pre erythrocytic stage, the authors conclude that the non BCG treated group showed no inflammation. Can this really be said? The number of parameters examined was very small. Please note that transcriptomic analysis of PBMC might have shown otherwise. 3) Figure 3C is not only negative data, but is predictable. I would drop it. 4) statements concerning the acquisition of anti-CSP antibodies seem rather trivial in nature. The authors are probably the world's experts on this topic, and should be aware that one challenge with 5 mosquitoes would not be likely to result in acquired immunity to sporozoite challenge. (as an aside, it would be nice if the authors included in the Methods section of the paper a little bit of data about what the actual challenge was. What percentage of their mosquitoes are actually infected and how many sporozoites per mosquito were expected as an infecting dose. How variable is sporozoite challenge? This may be in their earlier reports, but a short

statement would be useful). 5) lines 144-146. Similar comments about making a sweeping statement about the lack of inflammation 6) lines 179-181. Is there already clinical data on the effects of BCG on surviving malaria? It seems that given the clinical studies published to date, some knowledge is already publicly available. 7) line 209-211. I do not understand what this means. The authors are using the word "allocation" in a way that is foreign to a native English speaker. Please re write the sentence. 8) Figures: I dno not understand why some of the figures are only showing data in the BCG group and not the controls. For example, 1D-F and Fig 2. In addition, I think the legends are a bit sparse in their descriptions of the experiments. I am not certain if this is because of a word count requirement, but they could be a little easier to understand.

Response to reviewers' comments:

We would like to thank the reviewers for their review and constructive comments. We feel this has given us the opportunity to significantly improve the manuscript. We have responded to the reviewers' remarks point-by-point below, and have indicated with tracked changes where the manuscript has been changed.

Sincerely yours on behalf of all authors,

Prof. Robert W. Sauerwein

Manuscript ID: NCOMMS-18-02828

Reviewer #1 (Remarks to the Author):

The authors show that BCG vaccination 5 weeks before controlled human malaria infection results in better control of parasitaemia and increased activation of NK cells and monocytes coinciding with the appearance of blood stage parasites in peripheral circulation. Due to the nature of the experiments, the number of volunteers are small and the changes are observed only in approximately half of the BCG-immunised volunteers but nevertheless significant. The increases in NK and monocyte activation appear to be due to a heightened reactivity of these cells rather than persistent activation and as such form part of the recently described innate or trained memory. The results are interesting and novel in that the effect of BCG vaccination on human malaria have not been investigated previously. However, the mechanisms underlying the increased responsiveness of NK cells after BCG vaccination are not (yet) known or why they occur only in a proportion of BCG vaccinated volunteers.
Furthermore, given that BCG vaccination in infancy is part of the EPI programme in Sub-Saharan Africa, benefits for immunity to malaria or changes in vaccination regime to deploy such benefits seem unlikely.

<u>Authors' response</u>: The most important novelty of the current study is the evidence that monocyte and NK cell responses after BCG vaccination correlate with decreased parasitaemia in a major clinically relevant infection. So far, BCG induced trained innate immune responses have been confined to in vitro cell stimulation studies where the underlying cellular mechanisms have been extensively explored by us and others (Arts et al. Cell Host and Microbe 2017; Chen Cheng et al. Science 2014; Saeed et al. Science 2014). Indeed, these studies were restricted to transcriptomic and epigenetic changes in monocytes rather than NK cells which clearly warrants further study in future trials. We have highlighted this in the discussion:
<u>Text added to the manuscript:</u> '*A recent study examined the epigenetic and transcriptomic changes in monocytes of healthy volunteers vaccinated with BCG (Arts et al. Cell Host and Microbe 2018) , showing genome-wide changes in histone H3 acetylation at lysine 27 (H3K27ac) in 'responding' volunteers. Our study finds functional changes in NK cells as well, confirming previous in vitro observations (Kleinnijenhuis Clin Immunol 2014). This may be the result of increased monocyte activation, as NK cell activity against malaria is partially dependent on monocytes (Artavanis-Tsakonas, J Immunol 2003). Whether BCG induces epigenetic changes in NK cells as well should be subject of a future study.*'

As for malaria endemic countries where BCG is already part of the EPI program, these findings may a strong additional impetus to improve current BCG immunization practices and/or policies, particularly in areas where the incidence of tuberculosis is low.

The potential effect of BCG on malaria in endemic areas needs further study. A previous study showed that BCG revaccination did not reduce malaria morbidity (Rodrigues et al TMIH, 2006). However, this study did not take into account the effects of DTP vaccination during the study period, known to interfere with the overall non-specific effects of BCG (Roth et al. BMJ 2010). We have added the following text to the discussion.
Text added to the manuscript: *'Though BCG vaccination is common practice in malaria endemic countries as part of the WHO Expanded Programme on Immunization, potential efficacy against malaria and other pathogens underscores the need for investment in timely and correct BCG administration. Epidemiological data and randomized trials suggest revaccination with live-attenuated vaccines such as BCG confers additional protection against all cause mortality (Benn et al, EBioMedicine 2016). It will be important to determine whether BCG revaccination induces non-specific beneficial effects against malaria. Although BCG revaccination did not reduce malaria morbidity in one study in Guinea-Bissau (Rodriguez et al, TMIH 2007) potential confounding effects of other vaccines, including DTP with known interference with the overall non-specific effects of BCG (Roth et al, BMJ 2010) was not taken into account.'*

Minor comment 1:
Figure 1 and 2: The same graphs showing changes in platelet, lymphocyte and neutrophil counts (fig 1) as well as changes in leukocyte activation and cytokine levels (fig 2) should be shown for non-BCG vaccinated volunteers.

Authors' response: In figure 1D-F and Figure 2A-J data from the control group volunteers is shown as a box-plot, allowing direct comparison with the BCG vaccinated volunteers. We have clarified this in the legend of the figures.
Text added to the manuscript: *'Graphs show relative changes compared to pre-challenge values in both BCG vaccinated (each colored dot shows and individual volunteer) and non-BCG vaccinated controls (grey box-and-whiskers show median and Tukey's boxplot).'*

Minor comment 2:
The authors state in the discussion that BCG vaccination may have implication for improving immunity to malaria but do not further elucidate how that can be achieved. To my knowledge - almost all infants born in sub-Saharan Africa will be immunised with BCG at or shortly after birth and any beneficial effects on severe malarial disease at least during infancy - if the benefits of BCG vaccination last long enough as the authors seem to think - should be present already. Maybe the authors could expand how they see BCG vaccination deployed?

Authors' response: Though improvements in the timing and correct administration of BCG vaccine may still be made in malaria endemic areas, the greatest gains may be obtained where BCG revaccination prior to the malaria transmission season may improve protection or the acquisition of immunity.

Reviewer #2 (Remarks to the Author):
This manuscript describes a human malaria CHMI study using the standard 5 mosquito-bite model to induce falciparum malaria. This model is usually used to assess the efficacy of vaccines against malaria, however in this study the investigators have assessed the effect of BCG vaccination on immunity to malaria. The rationale for this innovative study is well-described in the introduction and is to assess the impact of inducing innate immunity on protection from malaria.

Major comment 1:
Unfortunately, while the rationale for the study is well-presented, the results are not and is most cases are either non-significant or only marginally significant. In addition, many of the claims made, particularly in the abstract are not supported by the data presented.

Firstly, the authors claim that the BGC-vaccinated participants experienced earlier and more severe clinical symptoms to malaria CHMI, but absolutely no detail is provided as to what these symptoms were or how they were graded and whether these symptoms were reported by participants themselves or observed by investigators (or whether either participants or investigators were blinded). As a consequence, one cannot determine the importance of this observation.

Authors' response: We have added detail about the collection and grading of adverse events to the manuscript. In short, both solicited and unsolicited adverse events were collected using patient diaries and daily questioning by the investigators. Adverse events were graded according to predefined criteria listed in the Clinical Trial Protocol.

As reported, the BCG vaccinated group as a whole developed earlier and more severe symptoms that the unvaccinated controls. We did not perform further sub-analyses per to symptom type because of the small number of volunteers. Moreover, the types of adverse events occurring after CHMI have been extensively published previously (Roestenberg et al. NEJM, 2009; Roestenberg et al. PlosOne 2012).

Text added to the manuscript:

'*Recording of adverse events*

Subjects recorded clinical symptoms in a diary, from the time of BCG vaccination until 37 days after the CHMI, as described previously (Roestenberg et al., NEJM, 2009; Roestenberg et al., PLOSOne 2012). Both solicited and unsolicited adverse events were recorded after questioning by the investigators at set time points: prior to BCG vaccination, prior to the CHMI, daily from day 6 after infection until 3 days after antimalarial treatment, and on day 37 post CHMI. Adverse events were graded according to criteria defined in the Clinical Trial Protocol: Mild (grade 1): awareness of symptoms that are easily tolerated and do not interfere with usual daily activity; Moderate (grade 2): discomfort that interferes with or limits usual daily activity; Severe (grade 3): disabling, with subsequent inability to perform usual daily activity, resulting in absence or required bed rest. Relatedness was assessed by the investigator, also on the bases of pre-defined criteria: Probable: An adverse event that follows a reasonable temporal sequence from the challenge procedure and cannot be reasonably explained by the known characteristics of the subject's clinical state; Possible: An adverse event for which insufficient information exists to exclude that the event is related to the study procedure; Not related: An event for which sufficient information exists to indicate that the aetiology is unrelated either because of the temporal sequence of events or because of the subject's clinical state or other therapies.'

Major comment 1 (cont):

Secondly, they claim that these symptoms were associated with the induction of the immune responses that they describe in the BCG-vaccinated group, but no evidence to support this association is presented and this association might be coincidental, particularly given the small numbers in this study.

Authors' response: It is very unlikely that this increase in moderate/severe symptoms in the BCG vaccinated group is coincidental. Severe symptoms (requiring bedrest) occurred in 4/9 volunteers in the BCG vaccinated group, which is indeed remarkably high and substantially deviates from historical data. Across all combined CHMI studies at our center where treatment was initiated at 100 Pf/mL as in this study the incidence was 3/42 CHMI control volunteers (unpublished).

The early and increased adverse events are present across the BCG vaccinated group. It is not possible to perform a proper statistical analysis to test association with inflammation considering the small number. However early symptoms (day 6, grade 1, 2 or 3) were seen in 4/4 BCG vaccinated volunteers with increased inflammation, and severe symptoms (grade 3) were seen in 3/4 BCG vaccinated volunteers with increased inflammation. This has been clarified in the abstract.

Text added to the manuscript: 'BCG vaccinated volunteers reported earlier and more severe clinical symptoms and had heterologous, memory-like monocyte and (innate) lymphocyte re-activation that correlated with reduced parasitemia at 5 weeks post vaccination.'

Major comment 1 (cont):
Thirdly, the author suggest that both the enhanced symptoms and induced immunity are associated with reduced parasitaemia at 5 weeks post CHMI, but again there is absolutely no analysis presented to support this. I couldn't even find the PCR data at 5 weeks in the manuscript.

Authors' response: The statement in the abstract that BCG vaccination reduces parasitemia at 5 weeks post CHMI, is indeed an error and has been corrected. The sentence has been changed to: 'BCG vaccinated volunteers reported earlier and more severe clinical symptoms and had heterologous, memory-like monocyte and (innate) lymphocyte re-activation that correlated with reduced parasitemia at 5 weeks post vaccination.'

Major comment 1 (cont):
Overall, the findings are weak and add little to the field. The trial is so poorly described that it is hard to determine the importance of the findings in the context presented. If the paper were re-written to describe the study to the standard usually expected, the findings would still be too marginal to be of importance. Most of the immunology figures only show the data for the BCG group, although the text describes comparisons with the unvaccinated group. Both ought to be shown to allow the reader to assess the validity of the claims made.

Authors' response: We clearly disagree with the reviewer. Data from this study, small and exploratory as it is, provide already important findings with potential field impact: 1) BCG vaccinated volunteers with an altered immune response are distinct from both BCG non-responders and controls across a number of relevant parameters, and 2) there are already strong correlations between altered immune responses and parasitemia. In our opinion, these clear findings in already a small cohort form a firm basis for required further confirmation in larger study groups.
The reviewer also notes that the immunology data should be shown for the control group as well. In figure 1D-F and Figure 2A-J data from the control group volunteers are shown as a box-plot, allowing direct comparison with the BCG vaccinated volunteers. We have clarified this in the legend of the figures.
Text added to the manuscript: *'Graphs show relative changes compared to pre-challenge values in both BCG vaccinated (each colored dot shows and individual volunteer) and non-BCG vaccinated controls (grey box-and-whiskers show median and Tukey's boxplot).'*

Major comment 1 (cont):
The study is also largely unreproducible as reported, particularly as the primary endpoint is not described nor the method used to assess it. No information is given about the method for the primary endpoint of the trial (malaria qPCR) and whether the interpretation of PCR data was made by investigators blinded to the vaccination status of the volunteers. It's not stated how often or for how long PCR was performed. One assumes that PCR was performed in real-time, but again this is not described.

Authors' response: The qPCR was performed in real-time, once daily from day 6 after challenge until day 3 post antimalarial treatment, according to previously published protocols. This has been added to the methods section.
Text added to the manuscript: *'qPCR was performed prospectively, once daily from day 6 after CHMI until day 3 after anti-malarial treatment, according to previously published protocols (Hermsen et al. Mol Biochem Parasitol 2001; Schats et al. PlosOne 2015; Walk et al. Malaria J 2015).'*

Major comment 1 (cont):

There is no CONSORT checklist and the T cell methods do not meet the MIATA guidelines, particularly for the ICS itself where the method is minimally described despite ample space in the supplementary to do this properly. The BCG dose is not specified.

Authors' response: Many of the items on the CONSORT checklist (blinding, interim analysis etc.) are not applicable to this study. Furthermore, as this was an exploratory study to answer basic immunological questions, it was not possible to completely pre-define all secondary outcome measurements nor calculate a sample size for each secondary outcome prior to study start.
Detailed method information is provided for each immunology assay including supplier for each reagent and antibody used. The culture medium for the T cell ICS assay has been added as well as the specifics of the flow cytometer. We present the gating strategy for a representative sample but feel that complete reporting of the T cell raw data (according to MIATA recommendations) will be overdone given the relative contribution of these data to the overall message of the paper.
Text added to the manuscript: *'Cells were cultured in RPMI 1640 (Dutch Modification; Gibco) with 5mg/ml gentamycin (Centraform), 100mM pyruvate (Gibco), 200mM glutamax (Gibco), supplemented with 10% heat-inactivated pooled human A+ serum (obtained from Sanquin Bloodbank, Nijmegen, The Netherlands).'*
'Samples were analyzed on a Gallios flow cytometer (Beckman Coulter) the same day. Flow cytometry data was analysed using Flow Jo software (version 10.0.8 for Apple OS). CD107a and cytokine responses to PfRBC were corrected for uRBC at every time point (thus, defined as percent increase over background), and then corrected for baseline (pre-vaccination) responses.'

BCG vaccination was administered at standard dose.
Text added to the manuscript: *'Ten subjects received standard dose (0.1mL of the reconstituted vaccine) of intradermal BCG vaccination (BCG Bulgaria, Intervax) five weeks prior to challenge infection.'*

Major comment 1 (cont):
The statistical analyses presented are appropriate, although often described in the text and not on the figure itself. Also, it would be useful to know where if a statistic is not given whether a test was performed and a non-significant result obtained and whether correction for multiple analyses were made. A Kaplan-Meier analysis should be performed for the qPCR data during follow-up.

Authors' response: Generally, we did not perform broad statistical analyses on each time point given the small study size. Therefore the outcome of the tests not always presented on the figures. We performed statistical analysis of the qPCR Kaplan-Meier curve (figure 1A) and added the p-value to the figure (non significant).

Reviewer #3 (Remarks to the Author):
This is a very interesting study about trained innate immunity in a malaria CHIM, and the authors make a fair case that this can exist. I am generally favorable to the report, although I think that there were some fundamental flaws in study design that cannot be corrected given the effort that is required for CHIM to be undertaken. First and foremost was the relatively short interval between BCG vaccine and challenge. The clinical observations surrounding trained innate immunity occur on a much longer time scale, so 5 weeks seems like a very short time interval between vaccine and challenge.

Authors' response: Designed as proof-of-concept study for potential innate effects, we deliberately choose for a relatively short interval between BCG and malaria i.e. before a person may have acquired adaptive immune responses through exposure. Previous studies with *ex vivo* cell restimulation have shown that the effect is detectable as from 2 weeks post vaccination. Studies on BCG vaccination in

mice show effects on malaria infection at 1-2 months post vaccination. Based on this data we selected 5 weeks as a clinically relevant time point. However, we agree with the reviewer that follow-up studies should investigate its effects against malaria over a longer time window, as effects on innate immunity can persist up to one year after BCG vaccination (Kleijnijenhuis, PNAS, 2012).

Reviewer #3 (cont):
My second objection to the trial design was the very very short period when parasitemia was tolerated. As parasitemia was always sub patent, there was very little to suggest that at least one more day of clinical illness was dangerous to the volunteers. I realize, of course, that Sauerwein and colleagues were probably told by their institutional ethics panel how much clinical illness was acceptable, but this decision contributes to what appear to be significant but small effects that run through the manuscript.

Authors' response: As these studies are conducted in healthy volunteers, the ethical board, indeed, requires us to use very stringent safety criteria. As suggested by the reviewer, a follow-up that would include at least a slightly longer study period allowing to see potential effects on blood stage parasite replication, would be of great value. While we see already clear effects on pre-erythrocytic stages, we may consider in a next study to use the established malaria blood stage challenge model administrating a very small inoculum intravenously thereby allowing a number of parasite replication cycles before curative treatment is initiated.

Reviewer #3 (cont):
A third deficiency, and one that may actually be addressable even at this time point, is the lack of transcriptomic data as well as ChIP seq data. Such data, while expensive and (in the case of ChIP seq) labor intensive, would greatly increase the impact of the manuscript.

Authors' response: We recently published data on epigenetic changes after BCG showing genome-wide changes in histone H3 acetylation at lysine 27 (H3K27ac) in monocytes one month after BCG vaccination (Arts et al. 2018 Cell Host and Microbe). Analysis of 646 differential peaks (baseline vs. 1 month after vaccination) showed changes in the regulation of several important signaling and inflammatory pathways. Moreover, differences were found in H3K27ac between BCG-responders and non-responders. Although the main message of this paper focuses on clinical parasitological effects, confirmation of these findings could indeed add value and benefit our present dataset. However, due to limitations in samples for analysis, we will be unable to draw tangible conclusions. In 3 BCG vaccinated volunteers representing 1 non-responder and 2 responders we found ≥2-fold changes in H3K27ac in 40 regions following vaccination, at least indicating that epigenetic changes do occur. Some of these regions overlap with the promoter/enhancer of several important genes in immune response, such as NCF2, IFIT5, MR1. However, further studies are obviously needed to specifically accommodate this valuable suggestion of the reviewer.
Text added to the manuscript: '*A recent study examined the epigenetic and transcriptomic changes in monocytes of healthy volunteers vaccinated with BCG (Arts et al. Cell Host and Microbe 2018) , showing genome-wide changes in histone H3 acetylation at lysine 27 (H3K27ac) in 'responding' volunteers. Our study finds functional changes in NK cells as well, confirming previous in vitro observations (Kleinnijenhuis Clin Immunol 2014) . This may be the result of increased monocyte activation, as NK cell activity against malaria is partially dependent on monocytes (Artavanis-Tsakonas, J Immunol 2003). Whether BCG induces epigenetic changes in NK cells as well should be subject of a future study.*'

Minor comment:
All of this being said, this is a piece of work that has importance.
Some minor comments:

1) why do the authors believe that BCG vaccinate patients were more symptomatic? This seems like a fairly robust observation, but the numbers are small and the explanation is not obvious.

Authors' response: The observed increase in symptoms may be related to the activated inflammatory response. Four BCG vaccinated volunteers reported grade 3 (severe) clinical symptoms during the trial. Interestingly, all four had early increases in IFN-γ or granzyme B and an early increase in CRP (either day 5 or day 7 post CHMI).

Minor comment 2:
2) during the pre erythrocytic stage, the authors conclude that the non BCG treated group showed no inflammation. Can this really be said? The number of parameters examined was very small. Please note that transcriptomic analysis of PBMC might have shown otherwise.

Authors' response: We have included the reviewer's note to our discussion.
Text added to the manuscript: '*More sensitive techniques such as (single-cell) transcriptomic analysis may be needed to study peripheral blood responses during the liver stage*.'

Minor comment 3:
3) Figure 3C is not only negative data, but is predictable. I would drop it.

Authors' response: We removed figure 3C, and changed the statement in the results to 'data not shown'.

Minor comment 4:
4) statements concerning the acquisition of anti-CSP antibodies seem rather trivial in nature. The authors are probably the world's experts on this topic, and should be aware that one challenge with 5 mosquitoes would not be likely to result in acquired immunity to sporozoite challenge.

Authors' response: We agree with the reviewer and have adapted the text accordingly.
Text added to the manuscript: '*However, a CHMI with 5 mosquito bites is not likely to induce significant cellular or humoral immunity, and this hypothesis should be tested in a study combining BCG with a malaria vaccine.*'

Minor comment 5:
as an aside, it would be nice if the authors included in the Methods section of the paper a little bit of data about what the actual challenge was. What percentage of their mosquitoes are actually infected and how many sporozoites per mosquito were expected as an infecting dose. How variable is sporozoite challenge? This may be in their earlier reports, but a short statement would be useful).

Authors' response: We have included a table with details of the challenge (supplementary table 2).

Supplementary table 2 added to the manuscript:

	**Mosquito infectivity**		**Infection**		
	Percent	# Sporozoites per mosquito	Number of sessions median (range)	# Infected bites median (range)	# Uninfected bites median (range)

BCG group	100%	160,500	1 (1-3)	5	0 (0-1)
Control group			1 (1-2)	5	0 (0-1)

*‘**Supplementary table 2**: **Data on mosquito infection and controlled human malaria infection.** All volunteers were infected with the same batch of P. falciparum infected Anopheles stephansi mosquitoes. Batch infectivity and mean sporozoite load was determined by dissection of a sample of 10 mosquitoes one day before the challenge infection. All volunteers received exactly 5 bites from infected mosquitoes. Most volunteers required only one or two sessions for a sufficient number of infected mosquito bites, with a single exception who required a third session.’*

Minor comment 6:
5) lines 144-146. Similar comments about making a sweeping statement about the lack of inflammation

Authors’ response: We agree with the reviewer that our analysis of immune cell phenotype and circulating cytokines does not completely exclude persistent inflammation that might be detectable with sensitive transcriptomic approaches. However, this statement refers to the fact that the increased immune activation (phenotype) and cytokine production seen in the BCG vaccinated volunteers after infection was not present before the malaria challenge, and therefore does not represent persistent activation. We have rewritten the statement to clarify this.
Text added to the manuscript: ‘*This prompt re-activation of immune responses in BCG vaccinated volunteers apparently represents a true memory phenotype rather than persistent inflammation, as prior to CHMI there was no difference in activation of peripheral blood leukocytes or circulating cytokine levels between the control and test groups.*’

Minor comment 7:
7) lines 179-181. Is there already clinical data on the effects of BCG on surviving malaria? It seems that given the clinical studies published to date, some knowledge is already publicly available.

Authors’ response: Most studies on the clinical, non-specific effects of BCG vaccine focus on all cause mortality. Subgroup analyses that look at malaria in these studies are difficult to interpret as they are often based solely on patient histories. We have added a brief note to the discussion.
Text added to the manuscript: ‘*There is limited data on BCG and the incidence of malaria from observational studies, with one study showing a reduction in malaria mortality in BCG vaccinated infants (Roth et al. Int J Epidemiol 2005*).

Minor comment 8:
8) line 209-211. I do not understand what this means. The authors are using the word "allocation" in a way that is foreign to a native English speaker. Please re write the sentence.

Authors’ response: We have changed the sentence.
Text added to the manuscript: ‘*Subjects and investigators were not blinded, whereas those performing the qPCR analysis were blinded until after the last qPCR data had been collected.*’

Minor comment 9:
8) **Figures: I do not understand why some of the figures are only showing data in the BCG group and not the controls. For example, 1D-F and Fig 2. In addition, I think the legends are a bit sparse in their descriptions of the experiments. I am not certain if this is because of a word count requirement, but they could be a little easier to understand**.

Authors' response: In figure 1D-F and Figure 2A-J data from the control group volunteers are shown as a box-plot, allowing direct comparison with the BCG vaccinated volunteers. We have clarified this in the legend of the figures, and have re-written the legends for figure 1 and 2 to make them easier to understand.

Text added to the manuscript:

*'**Figure 1**: **parasitemia, clinical symptoms and laboratory abnormalities after Controlled Human Malaria Infection.** Parasitemia was measured by daily qPCR from day 6 after CHMI until the third day after antimalarial treatment. **(A)** The Kaplan-Meier survival curve shows percent of volunteers remaining untreated. 8/9 BCG vaccinated (green) and 10/10 control volunteers (grey) surpassed the treatment threshold of 100 parasites per milliliter, and were treated on day 7 after challenge. 1/9 BCG vaccinated volunteers remained below 100 Pf/mL until day 9**.** **(B)** All volunteers did have parasitemia detectable by qPCR on day 7 after CHMI. The graph shows log parasites per milliliter on day 7 post CHMI for BCG vaccinated (green) and control (grey) volunteers. There was greater heterogeneity in parasitemia levels in the BCG vaccinated volunteers compared to controls. **(C)** Adverse events were collected daily. The Kaplan-Meier curve shows the percentage of volunteers experiencing one or more moderate or severe, solicited, symptoms during follow-up. BCG vaccinated volunteers (green) experienced earlier and more moderate/severe symptoms than controls (grey). **(D-F)** Absolute platelet, lymphocyte and neutrophil differentiation counts were determined by daily hemocytometry starting on day 6 post challenge. Graphs show relative change in cell counts compared to pre-challenge values in both BCG vaccinated (each colored dot shows and individual volunteer) and non-BCG vaccinated controls (grey box-and-whiskers show median and Tukey's boxplot). '*

*'**Figure 2: in vivo activation of lymphocytes, monocytes and neutrophils, and cytokine production after Controlled Human Malaria Infection.** In vivo leukocyte activation was determined by direct staining of fresh whole blood with fluorescent antibodies every two days post challenge. Lymphocytes were defined based on forward scatter and sideward scatter characteristics, and duplet events were excluded. **(A)** NK cell activation was defined as the percentage of $CD3^{-}CD56^{dim}CD16^{+}$ live cells expressing CD69. **(B)** γδT cell activation was defined as the percentage of $CD3^{+}\gamma\delta TCR^{+}$ live cells expressing CD69. **(C)** NKT cell activation was defined as the percentage of $CD3^{+}\gamma\delta TCR^{-}CD56^{+}$ live cells expressing CD69. **(D)** αβT cell activation was defined the as percentage of $CD3^{+}\gamma\delta TCR^{-}CD56^{-}$ live cells expressing CD69. **(E)** Monocytes were defined based on forward and side scatter characteristics, and then as $HLA\text{-}DR^{+}CD14^{+}$. Within the monocyte population, cells were then divided into $CD16^{-}$ and $CD16^{+}$ monocytes. **(F-G)** Within the $CD16^{-}$ monocyte population, the relative change in mean fluorescent intensity of HLA-DR and CD86 compared to pre- malaria challenge values was determined. **(H)** Neutrophils were defined based on forward and side scatter characteristics, and the defined as $HLA\text{-}DR^{-}CD14^{-}CD16^{+}CD11b^{+}$. Activated neutrophils were defined as $CD62L^{dim}CD11b^{high}$. **(I-J)** IFN-γ and granzyme B were measured by Luminex assay in citrate plasma taken ever two days. Circulating cytokine levels are corrected for baseline levels (pre-BCG vaccination time point) at each time point. In all graphs the grey box-and-whiskers show the median and Tukey's boxplot of non-BCG vaccinated control group volunteers, and each colored dot shows an individual BCG vaccinated volunteer. **(K)** Circulating CRP levels were measured in citrate plasma are shown for each BCG vaccinated volunteer (colors consistently represent the same volunteers across each graph).'*

REVIEWERS' COMMENTS:

Reviewer #2 (Remarks to the Author):

The revised manuscript has improved a lot and my points were addressed satisfactorily. However, prior to publication the following points must be addressed:

1. Figure legends in general: The authors should always indicate how many mice were analyzed and how many independent experiments were performed. In some legends this information is missing.
2. Legend Fig. 4 (F): The authors state “Compiled MFIs from three independent experiments are shown (F).“ According to the Source Data file only 2 experiments were performed.
3. Legend Fig. 4 (I): The authors state “Data were compiled from three independent experiments.“ According to the Source Data file only 2 experiments were performed.
4. Please clarify why some of the values related to Fig. 2F and Fig. 2H (see Source Data files) are identical although different parameters were analyzed. The same is true for the values related to Fig. 5D and Fig. 5F.

Reviewer #3 (Remarks to the Author):

The authors did an outstanding job of responding to the reviewers' concerns and altering their manuscript appropriately.

I note that two reviewers thought that mixed bone marrow chimeraes would be very useful to verify the proposed mechanism. If they can't be made, is there a way to acknowledge their utility?

Response to editor's and reviewers' comments:

We would like to thank the editor and the reviewers for their constructive comments and the chance to revise the manuscript. We have responded to the remarks point-by-point below, and have indicated with tracked changes where the manuscript has been changed.

Sincerely yours on behalf of all authors,

Prof. Robert W. Sauerwein

Reviewers' comments:

Reviewer #1 (Remarks to the Author):

The authors addressed all my concerns

Reviewer #2 (Remarks to the Author):

The manuscript has been revised and the additional information does improve the overall readability and validity of the findings. However, my major comment has not been addressed. There is still no detail provided on the enhanced, early clinical symptoms observed in the vaccinated cohort. It is very hard to argue that BCG vaccination could confer an improved response to the infection due to reduced parasitaemia, whilst simultaneously demonstrating enhancement of the presentation of clinical malaria.

Author's response: We agree that based on this study we cannot make the claim that BCG vaccination improves the clinical course or mortality of a natural malaria infection. However, this is the not claim made in the manuscript. Instead, we are referring to an improved immune response against parasitemia, focusing on the functional activity of trained immunity against malaria parasites.

Reviewer #2 (Remarks to the Author):

It is therefore of substantial interest to the reader to understand how vaccination has changed the clinical presentation. For example, in the rebuttal, the author's state that 4/9 vaccinees required bedrest after CHMI compared with 3/42 on average previously. This ought to be reported in the text as should the enhancement or otherwise of other systemic symptoms, in particular fever. The observation of moderate/severe symptoms in 80% of vaccinated volunteers (compared with 30% in the unvaccinated) is striking to say the least and merits discussion.
In most cases, enhancement of clinical presentation would not be considered a positive for a vaccine candidate.

Author's response: We find that BCG vaccination increased the frequency of all symptoms typically associated with Controlled Human Malaria Infection, including headache, gastro-intestinal symptoms and systemic symptoms like fatigue and myalgia. This data has now been added as supplementary table 2. Significant fever is rare in CHMI when treatment is initiated early and is seen in only a few volunteers in this study. There was no difference in temperature between the BCG vaccinated and control volunteers, this information has been added to the supplementary materials (supplementary figure 2).

In addition, we have added data on the incidence of moderate and severe adverse events in volunteers participating in other CHMIs at our center if the same parasite strain and treatment criteria were used (data from 35 volunteers met these criteria). This data has been added to supplementary figure 6.

Text added to the manuscript (changes underlined):
(Methods)
'<u>Oral temperature was measured by volunteers and recorded in the symptom diary every morning and more frequently during symptoms. Tympanic temperature was measured by the study physician at every follow-up visit. Fever was scored as follows: mild (grade 1): 37.6-38.0° Celsius; moderate (grade 2): 38.1-39.0° Celsius; severe (grade 3): ≥39.1° Celsius.</u>'
(Results)
'BCG-vaccinated volunteers developed clinical symptoms of malaria infection at an earlier time point and reported a higher frequency of moderate or severe clinical symptoms than control volunteers (p=0.01, figure 1C; <u>supplementary table 2</u>).'
'<u>There was no significant difference in temperature during follow-up (supplementary figure 2).</u>'
(Discussion)
'In addition, the course of clinical symptoms is strikingly different from <u>that seen in other, similar CHMI studies at our center (supplementary figure 6),</u> where symptoms are typically absent on day 7 post-challenge.'

Reviewer #2 (Remarks to the Author):
I also pointed out previously that there was no data supporting the statement that BCG vaccination reduced parasitaemia and while this statement has been removed from the abstract, the results (line 109-110) still state that BCG-induced immunity correlates with lower parasitaemia. While the regression was highly significant for CD69 expression of NK's, the second correlation with HLA-DR is much less convincing.

<u>Author's response</u>: As we also detailed above in the response to the editor, we have toned down the text regarding the impact of the BCG vaccination on parasitemia, and describe more clearly that this effect occurs in a subgroup of individuals. We do not make the claim that BCG vaccination reduced parasitemia on group level. Instead we make the case that the rapid innate immune responses seen in a subset of vaccinated volunteers correlate with decreased parasitemia. We have clarified this distinction in the revised manuscript.

Text added to the manuscript (changes underlined):
(Results)
'Indeed, <u>the subset of</u> BCG-vaccinated volunteers with early lymphocyte and monocyte activation were <u>also</u> those with lower parasitemia (figure 3A-B and supplementary figure 3), and early NK cell CD69 expression and monocyte HLA-DR expression were ~~strongly~~ correlated with decreased parasitemia.'
(Discussion)
'Interestingly, the earlier and stronger immune activation in half the BCG vaccinated volunteers <u>correlated with a reduced</u> parasitemia in early infection.'

Reviewer #3 (Remarks to the Author):

I am still positive on this manuscript, even after reading the reviews by reviewer #2. In general, the tone of the argument made by Walk et al. has been moderate, ie, no large sweeping claims are made. I am pleased that the authors have clarified much of the methodology as well as re written the figure legends to make data interpretation easier. I still find the manuscript very interesting and the data worthy of being reported.

That being said, I am a bit disappointed with the authors for not adding some language to the paper (as opposed to their rebuttal of my review) explaining why they chose a 5 week time point.

Author's response: We thank the reviewer for his/her positive assessment. We have added the information on the 5-week time period to the manuscript.

Text added to the manuscript (changes underlined):
(Discussion)
'For this study the observation period of five weeks was chosen based on evidence of BCG induced protection against malaria in mice at 1-2 months post vaccination and BCG induced trained innate immunity in humans at 2 weeks and 3 months post vaccination.'

Reviewer #3 (Remarks to the Author):

I actually do not understand their response to my objections concerning the degree to which parasitemia was tolerated. I am not certain why a blood stream challenge model will be much different in terms of tolerated parasitemia than challenge by mosquito.

Author's response: In mosquito challenge studies at our center parasitemia reaches the levels detectable by thick blood smear at a mean of 10.5 days post challenge, 3.5 days after the first appearance of parasitemia (Walk and Schats et al. Malaria J 2015). In contrast, a blood stage inoculation of 1000 infected erythrocytes reaches the same treatment threshold after 8.5 days (Bijker and Bastiaans et al. PNAS 2013). We hypothesized that the longer parasitemia in the latter model would make it more sensitive in this case. We have clarified this in the Discussion.

Text added to the manuscript (changes underlined):
(Discussion)
'In future studies, this might be addressed by allowing longer duration of parasitemia, or alternatively, by using a blood stage challenge infection with a low inoculum, which would allow for even longer exposure to blood stage parasites.'

Reviewer #3 (Remarks to the Author):

Finally, I understand that they have reported epigenetic markers in BCG infected individuals in the past, the correlation of immune markers to parasitemia that they claim to have demonstrated might have been reflected by similarly altered ChIP seq results.

Author's response: We agree with the reviewer that an analysis comparing changes in immune phenotype during infection and epigenetic changes after BCG vaccination and during CHMI would be extremely interesting. Unfortunately, due to limitations in cellular samples available, there is currently insufficient ChIPseq data available for a meaningful analysis. This will be a very important question for future studies.
